# Psychological Determinants of Whole-Body Endurance Performance

**DOI:** 10.1007/s40279-015-0319-6

**Published:** 2015-03-15

**Authors:** Alister McCormick, Carla Meijen, Samuele Marcora

**Affiliations:** Endurance Research Group, School of Sport and Exercise Sciences, University of Kent, Medway Building, Chatham Maritime, Kent, ME4 4AG UK

## Abstract

**Background:**

No literature reviews have systematically identified and evaluated research on the psychological determinants of endurance performance, and sport psychology performance enhancement guidelines for endurance sports are not founded on a systematic appraisal of endurance-specific research.

**Objective:**

A systematic literature review was conducted to identify practical psychological interventions that improve endurance performance and to identify additional psychological factors that affect endurance performance. Additional objectives were to evaluate the research practices of the included studies, to suggest theoretical and applied implications, and to guide future research.

**Methods:**

Electronic databases, forward-citation searches and manual searches of reference lists were used to locate relevant studies. Peer-reviewed studies were included when they chose an experimental or quasi-experimental research design; a psychological manipulation; endurance performance as the dependent variable; and athletes or physically active, healthy adults as participants.

**Results:**

Consistent support was found for using imagery, self-talk and goal setting to improve endurance performance, but it is unclear whether learning multiple psychological skills is more beneficial than learning one psychological skill. The results also demonstrated that mental fatigue undermines endurance performance, and verbal encouragement and head-to-head competition can have a beneficial effect. Interventions that influenced perception of effort consistently affected endurance performance.

**Conclusions:**

Psychological skills training could benefit an endurance athlete. Researchers are encouraged to compare different practical psychological interventions, to examine the effects of these interventions for athletes in competition and to include a placebo control condition or an alternative control treatment. Researchers are also encouraged to explore additional psychological factors that could have a negative effect on endurance performance. Future research should include psychological mediating variables and moderating variables. Implications for theoretical explanations for endurance performance and evidence-based practice are described.

**Electronic supplementary material:**

The online version of this article (doi:10.1007/s40279-015-0319-6) contains supplementary material, which is available to authorized users.

## Key Points

**Table:** 

Practical psychological interventions consistently improve endurance performance in published studies. Psychological skills training could therefore benefit an endurance athlete. There is more to learn, however, about how (i.e. mediating variables) and for whom (i.e. moderating variables) these interventions work.
Verbal encouragement and head-to-head competition can have a beneficial effect on endurance performance and should be controlled in experiments.
Mental fatigue has a negative effect on endurance performance.

## Introduction

A systematic literature review was conducted to identify practical psychological interventions that improve endurance performance and to identify additional psychological factors that affect endurance performance. For the purpose of this review, endurance performance is defined as performance during whole-body, dynamic exercise that involves continuous effort and lasts for 75 s or longer (see next paragraph). Although single or combined running, cycling and swimming events (e.g. marathons, triathlons, ultra-marathons) are most often associated with endurance, other endurance sports could include rowing, canoeing, cross-country skiing and speed skating. Visual inspection of the performance times at the London 2012 Summer Olympics (http://www.olympic.org/sports) suggested that more than 70 events met our definition of endurance performance. Endurance sports are also popular with recreational participants. In 2014, for example, there were more than 35,000 finishers in the London Marathon [1] and more than 13,000 people participated in the London Triathlon [2]. Identification of psychological factors that have a causal relationship with endurance performance would support evidence-based practice. At present, however, no literature reviews have systematically identified and evaluated research on psychological determinants of endurance performance. Furthermore, in sport psychology, performance enhancement guidelines for endurance sports [3–8] are not founded on a systematic appraisal of endurance-specific research.

Sport psychology research on endurance performance can be divided into muscular endurance and cardiorespiratory endurance [9]. Muscular endurance tasks (e.g. sit-ups, weight holding, hand-grip tasks and leg-raise tasks) mostly involve a single muscle or muscle group [10]. In contrast, cardiorespiratory or aerobic endurance refers to “the entire body’s ability to sustain prolonged, dynamic exercise using large muscle groups” [10] (p. 223). This review focuses on aerobic endurance because it represents those whole-body endurance tasks that people perform recreationally and competitively. Physiologically, aerobic endurance relies primarily on energy that is derived from aerobic—as opposed to anaerobic—metabolism. The aerobic energy system produces large amounts of energy through combustion of carbohydrates and fats, but it produces energy at a slower rate than the anaerobic energy system [11]. The relative contribution of the aerobic energy system increases with the duration of maximum-effort exercise, and Gastin [11] estimated that the relative contribution of the aerobic energy system generally predominates after 75 s of maximum-effort exercise. As an eligibility criterion, endurance performance was therefore defined as performance during whole-body, dynamic exercise that involves continuous effort and lasts for 75 s or longer.

This review focuses on the psychological determinants of endurance performance. Whereas a correlate demonstrates a reproducible association or predictive relationship with a dependent variable, a determinant demonstrates a cause-and-effect relationship [12]. Correlates of endurance performance include a positive affect [13], self-efficacy [14], use of psychological strategies [15], personal-standards perfectionism, performance approach goals and self-set personal goals [16]. This systematic review aimed to support evidence-based practice by identifying practical psychological interventions and other psychological factors that have been shown to have a causal relationship with endurance performance in experimental or quasi-experimental research (i.e. psychological determinants).

Practical psychological interventions were defined as psychological manipulations judged to be ethical, feasible and accessible to a sport practitioner, coach or athlete. Although meta-analyses support use of goal setting [17], imagery [18], self-talk [19] and psychological skills training (PST) packages [19] to improve performance in a range of sport and exercise tasks, the effects of PST on endurance performance have not been reviewed. In contrast, associative and dissociative cognitive strategies have received much interest in the endurance literature (for reviews on this subject, see Brick et al. [9]; Brewer and Buman [20]; Lind et al. [21]; Masters and Ogles [22]; and Salmon et al. [23]). Much research on association and dissociation, however, is correlational or observational [22]; this review is interested in the experimental studies that have examined whether these cognitive strategies affect endurance performance. Although music, placebos, feedback and deception can be used to improve endurance performance, they were not included in the present review, because these psychological manipulations have been thoroughly and recently reviewed elsewhere [24–28].

Identification of practical psychological interventions that improve endurance performance, as well as additional psychological factors that affect endurance performance, could benefit the performance of competitive endurance athletes. Further, identifying methods for improving endurance performance could encourage recreational participants’ continued involvement in sport by increasing their self-efficacy [29] or perceived competence [30]. Although experimental and quasi-experimental studies have been examining the effects of psychological factors on endurance performance for nearly 50 years [31], the psychological determinants of endurance performance have not been reviewed systematically. A systematic literature review was therefore conducted to identify the psychological factors that have been shown to affect (or not affect) endurance performance and to evaluate the research practices of these studies. By synthesizing research on the psychological determinants of endurance performance, this systematic review aimed to inform theoretical perspectives of endurance performance, support evidence-based practice and guide future research.

## Methods

### Sources

Studies were identified by searching the following resources: (1) electronic databases (Academic Search Complete, PsycARTICLES, PsycINFO, Scopus and Web of Knowledge); (2) reference lists of included studies and other psychological research articles, review articles and book chapters on endurance performance and related topics (e.g. perception of effort, association and dissociation); and (3) forward-citation results in Google Scholar and Web of Knowledge. Academic Search Complete, PsycARTICLES and PsycINFO were searched together, using EBSCOhost. In total, 128 endurance-related keyword variations were included in database searches (see Appendix S1 in the Electronic Supplementary Material). All available publication years were searched up to December 2014. Depending on the database search options, keywords were searched in both article titles and abstracts. Because ‘endurance’ is relevant to many sports and experimental procedures, 12 separate database searches were conducted for keywords relating to the following: endurance performance and its measurement (e.g. time to exhaustion), physiological dependent variables that may be measured during endurance performance (e.g. economy, pacing, maximal oxygen uptake [$\dot {\text {V}}\text{O}$_2max_]), running (e.g. cross-country, marathon), cycling (e.g. ergometer), rowing (row OR rower OR rowers OR rowing), skiing, canoeing, kayaking, swimming, speed skating, triathlon and race walking. Keywords were separated by the OR operator. Results were narrowed using the AND operator, which was combined with 78 keywords related to psychological states (e.g. anxiety), cognitive strategies (e.g. self-talk) and other psychological manipulations (e.g. reward). In Scopus and Web of Knowledge, the results were narrowed by filtering relevant research areas and subject areas, respectively (e.g. physiology, psychology, sport sciences). If an individual search returned over 1,000 results in EBSCOhost, the results were narrowed by filtering articles that included the words ‘sport’, ‘exercise’ or ‘perform*’ in the whole text. Abstracts of returned articles were examined unless the article’s title was clearly inconsistent with the topic of this review. The full text was examined if the abstract indicated that the study might meet the eligibility criteria, if the abstract provided insufficient information or if the abstract was unavailable.

### Eligibility Criteria

Studies were included if they met the following criteria: (1) written in English language; (2) published in a peer-reviewed journal; (3) used an experimental or quasi-experimental research design; (4) chose athletes or physically active, healthy adults as participants; (5) used a psychological manipulation; (6) met our definition of endurance performance; and (7) measured performance time, distance, work completed, power output, peak power, peak velocity or competitive outcome as the dependent variable. When studies did not quote performance times, 200 m was classed as the shortest endurance distance in swimming and 800 m was classed as the shortest endurance distance in running; maximum-effort performances at and above these distances would consistently last longer than 75 s and therefore satisfied our definition of endurance performance. As this review was interested in endurance performance, studies were excluded if participants were asked not to offer their maximum effort in the endurance task. To support evidence-based practice, studies that compared practical psychological interventions without a within- or between-subject control were excluded because it was not possible to judge whether any intervention was beneficial compared with no intervention. Feedback, deception, music and placebos were not included, because these psychological manipulations have been reviewed previously.

### Evaluation of Study Quality

All included studies were evaluated using a modified version of the Effective Public Health Practice Project (EPHPP) Quality Assessment Tool for Quantitative Studies [32]. The application instructions were adjusted to increase the relevance of the tool to sport science research (see Appendix S2 in the Electronic Supplementary Material for the modified application instructions). Studies were assigned ‘weak’, ‘moderate’ or ‘strong’ ratings for the following components when they were judged to be applicable: study design, confounders, blinding, data collection methods, and withdrawals and dropouts. Judgments were made when the ‘correct’ rating was unclear. When studies were assigned no ‘weak’ ratings, they were assigned an overall rating of either ‘strong’ (50 % < ‘strong’ ratings) or ‘moderate’ (50 % > ‘strong’ ratings). Studies with one ‘weak’ rating were assigned an overall rating of ‘moderate’, and studies with two or more ‘weak’ ratings were assigned an overall rating of ‘weak’. Intervention integrity was also evaluated, but studies were not assigned a quality label for this component. The selection bias component of the tool was not applied, because it is common practice for sport science studies to recruit participants using advertising material and by approaching sport teams (studies are assigned a ‘weak’ rating for self-referral). Participants in all but one of the studies [33] were self-referred, and inclusion of the selection bias component would therefore have reduced the discriminative ability of the tool. To safeguard against data extraction bias, an external researcher independently evaluated a random selection of nine studies (20 %), using the modified tool [34]. The two researchers then critically discussed the application of the tool to each of these studies. The independent researcher agreed with the overall quality label assigned to all nine studies. An audit trail was also used to document the decision-making process for all included studies (available on request).

Additional evaluation criteria were also applied. Moderating variables, psychological mediating variables and the number of participants whose endurance performance changed were recorded when applicable [35]. The presence of the following study characteristics was recorded for practical psychological interventions: a placebo control condition; an intervention-adherence check when use of an intervention was not observable (e.g. cognitive strategy, intervention practised at home); a description of the qualifications or experiences of the person delivering the intervention; a social validity or consumer satisfaction measure [35]; and the number of measured performances after intervention withdrawal [35].

### Effect Sizes

Effect sizes were calculated when mean and standard deviation (SD) values were either reported in the manuscript or provided by the authors on request. Glass’s Δ value was calculated for within-subject group designs [36]. For pretest–posttest designs with a control group, Δ values were calculated using the formula recommended by Morris [37]. For these two designs, the most recent control test of performance was chosen to calculate the effect sizes. When group-design studies included two main endurance performance dependent variables (e.g. performance time and total power, multiple performance distances), mean effect sizes (weighted by sample size) were calculated [19]. For single-subject, multiple-baseline designs, effect sizes were calculated for each participant and then a weighted average (accounting for missed performance tests) was calculated for the intervention (see Beeson and Robey [38]). For three of these studies [39–41], performance times were not stated and could not be determined precisely using manuscript graphs. Enlarged graphs were therefore printed, and the vertical distances of the data points from the *X* axis were measured by ruler. Effect sizes were then calculated by replacement of performance times with the measured distances [38]. Small, moderate and large effect-size anchors are substantially higher in single-subject designs (e.g. 2.6, 3.9 and 5.8, respectively, in a non-sport context; see Beeson and Robey [38]) than in group designs (e.g. 0.2, 0.5 and 0.8, respectively; see Cohen [42]). Readers should take these differences into account when comparing effect sizes. To avoid reporting misleading effect sizes for single-subject, multiple-baseline designs, the percentage of non-overlapping data points (PND) was also calculated for each participant and mean PND scores are reported for each intervention. This percentage is the proportion of post-intervention performances that were better than the best control test of performance. Scores of 90 % suggest a very effective treatment, scores of 70–90 % suggest an effective treatment, scores of 50–70 % suggest questionable effectiveness and scores below 50 % suggest an ineffective treatment [43]. To calculate an overall effect size for an intervention, effect sizes from different studies were weighted by the respective sample size.

## Results

‘Psychological’ and ‘endurance’ are broad terms. A search strategy with high sensitivity and low specificity was therefore chosen [34], which led to excessive database returns (>30,000 non-unique returns). It was therefore unfeasible to represent the search strategy in a flow chart. Nevertheless, the full texts of 101 studies were assessed for eligibility, and 46 studies were included (see Table S1 in the Electronic Supplementary Material for the reasons for exclusion). Studies that used practical psychological interventions (*n* = 25) are presented separately from the additional psychological determinants (*n* = 21). Table 1 (practical psychological interventions) and Table 2 (additional psychological determinants) present the information that was extracted from the included studies and evaluated, including the assigned quality ratings. Table S2 in the Electronic Supplementary Material (practical psychological interventions) and Table S3 in the Electronic Supplementary Material (additional psychological determinants) provide an overview of each included study. A narrative synthesis of evidence was chosen because of the heterogeneity of the independent and dependent variables, study designs and participant competitive levels [34].

**Table 1:** Quality review and evaluated study information—practical psychological interventions

Study information			Barwood et al. [53]	Barwood et al. [48]	Blanchfield et al. [49]	Burhans et al. [60]	Caird et al. [63]	Donohue et al. [33]	Donohue et al. [61]	Hamilton et al. [64]	Jackson et al. [50]	Lindsay et al. [44]	Miller and Donohue [46]	Morgan et al. [54]	Okwumabua et al. [47]
Quality criterion	Details														
Study design	Label		M	S	S	S	M	W	S	W	M	W	S	S	M
Confounders	Identified		N	?	?	N	N	N	N	N	?	Y	Y	N	Y
	Controlled			N	Y						Y	N	N		N
	Label		S	M	S	S	S	S	S	S	S	M	M	S	W
Blinding	Assessor										R	R		R	
	Participants		R	R	R	R					R				
	Label		M	M	M	M	–	W	W	W	S	M	W	M	W
Data collection methods	Valid		Y	Y	Y	Y	Y	Y	Y	Y	Y	Y	Y	Y	Y
	Reliable		R				R								
	Label		S	M	M	M	S	M	M	M	M	M	M	M	M
Withdrawals and dropouts	Reported		N	N	N	N	N	Y	N	Y	N	N	N	Y	N
	Label		W	W	W	W	W	S	–	M	–	W	–	S	W
Intervention integrity	Consistency														
	Contamination								C				C		
	Co-intervention							C	C				C		
Overall label			M	M	M	M	M	W	M	W	M	W	M	S	W
Intervention adherence			N	½	Y	Y	Y	–	–	Y	–	Y	Y	N	Y
Moderators			N	N	N	Y	N	N	N	–	Y	–	Y	N	N
Possible psychological mediators			Y	Y	Y	Y	–	N	N	N	N	Y	N	Y	N
Targeted			Y	N	Y	N						Y		N	
Placebo control			Y	N	N	N	–	N	N	N	N	N	N	N	Y
Social validity/satisfaction			N	½	N	?	N	Y	Y	–	–	Y	Y	–	N

Study information			Patrick and Hrycaiko [39]	Post et al. [55]	Rushall and Shewchuk [65]	Saintsing et al. [56]	Scott et al. [58]	Sheard and Golby [45]	Tenenbaum et al. [59]	Thelwell and Greenlees [40]	Thelwell and Greenlees [41]	Theodorakis et al. [51]	Weinberg et al. [62]	Weinberg et al. [57]
Quality criterion	Details													
Study design	Label		W	W	W	M	W	W	–	W	W	M	W	M
Confounders	Identified		N	N	N	N	N	Y	N	N	N	?	N	N
	Controlled							N				N		
	Label		S	S	S	S	S	M	S	S	S	M	S	S
Blinding	Assessor							R						
	Participants			R		R		R	R					R
	Label		W	M	W	M	W	S	M	W	W	W	W	M
Data collection methods	Valid		Y	Y	Y	Y	Y	Y	Y	Y	Y	Y	Y	Y
	Reliable			R										
	Label		M	S	M	M	M	M	M	M	M	M	M	M
Withdrawals and dropouts	Reported		N	Y	N	N	N	Y	N	Y	Y	Y	N	N
	Label		W	S	W	W	W	S	W	S	S	S	–	–
Intervention integrity	Consistency							C						
	Contamination				C									
	Co-intervention				C								C	
Overall label			W	M	W	M	W	M	–	W	W	M	W	M
Intervention adherence			Y	Y	½	Y	Y	½	Y	Y	Y	Y	½	Y
Moderators			–	–	N	N	–	N	Y	–	–	N	N	N
Possible psychological mediators			N	N	N	N	N	Y	–	N	N	N	N	Y
Targeted								½						N
Placebo control			N	N	N	N	N	N	–	N	N	N	N	N
Social validity/satisfaction			Y	Y	½	N	Y	Y	–	Y	Y	–	Y	–

*½* somewhat, *C* concern identified relating to intervention integrity (left blank when concern not identified), *M* ‘moderate’ quality label, *N* no, *?* unclear, *R* ‘yes—reported in manuscript’ (left blank when ‘no’ or ‘not reported’), *S* ‘strong’ quality label, *W* ‘weak’ quality label, *Y* yes, – not applicable to the evaluated study

**Table 2:** Quality review and evaluated study information—additional psychological determinants

Study information			Bath et al. [73]	Blanchfield et al. experiment 1 [52]	Blanchfield et al. experiment 2 [52]	Bubb et al. study 1 [79]	Bubb et al. study 2 [79]	Chitwood et al. [70]	Corbett et al. [67]	Franks and Myers study 1 [80]	Higgs [66]	Hodgins et al. [78]	Hulleman et al. [71]
Quality criterion	Details												
Study design	Label		M	S	S	M	M	S	M	S	W	S	M
Confounders	Identified		N	N	N	?	?	N	N	N	N	N	N
	Label		S	S	S	M	M	S	S	S	S	S	S
Blinding	Assessor							R					
	Participants		R	R	R					R		R	R
	Label		M	M	M	W	W	M	W	M	W	M	M
Data collection methods	Valid		Y	Y	Y	Y	Y	Y	Y	Y	Y	Y	Y
	Reliable								R				R
	Label		M	M	M	M	M	M	S	M	M	M	S
Intervention integrity	Consistency									C			
	Contamination												
	Co-intervention												
Overall quality	Label		M	M	M	M	M	M	M	M	W	M	M
Possible psychological mediators			Y	Y	Y	Y	Y	N	N	Y	N	N	N
Moderators			N	N	–	Y	Y	Y	N	Y	Y	N	N

Study information			MacMahon et al. [76]	Marcora et al. [75]	Miller [82]	Moffatt et al. [69]	Pageaux et al. [77]	Peveler and Green [68]	Viru et al. [72]	Wagstaff [81]	Williams et al. [74]	Wilmore [31]
Quality criterion	Details											
Study design	Label		S	S	S	S	S	W	S	S	M	M
Confounders	Identified		N	N	N	N	N	N	N	N	N	N
	Label		S	S	S	S	S	S	S	S	S	S
Blinding	Assessor			R	?							
	Participants		R	R	?		R		R	R	R	
	Label		M	S	M	W	M	W	M	M	M	W
Data collection methods	Valid		Y	Y	Y	Y	Y	Y	Y	Y	Y	Y
	Reliable							R				R
	Label		M	M	M	M	M	S	M	M	M	S
Intervention integrity	Consistency					C			C			
	Contamination											
	Co-intervention											
Overall quality	Label		M	S	M	M	M	W	M	M	M	M
Possible psychological mediators			Y	Y	–	Y	Y	Y	N	Y	Y	N
Moderators			N	N	Y	Y	N	N	N	N	N	N

*C* concern identified relating to intervention integrity (left blank when concern not identified), *M* ‘moderate’ quality label, *N* no, *R* ‘yes—reported in manuscript’ (left blank when ‘no’ or ‘not reported’), *S* ‘strong’ quality label, *W* ‘weak’ quality label, *Y* yes, *?* unclear, – not applicable to the evaluated study

### Quality of Included Studies

Two studies were classed as strong in quality (4 %), 31 studies were classed as moderate (69 %), 12 studies were classed as weak (27 %) and one study was assigned ‘not applicable’. Eighteen studies (40 %) were judged to have chosen a strong design. Confounders were identified in four of the included studies (9 %); in these studies, data were collected at different races [44, 45], posttests were more competitive than pretests [46] or pre-existing groups demonstrated substantial differences in pretest performance [47]. In four studies, it was unclear if the groups were equivalent at pretest [48–51]; two of these studies, however, controlled for pretest performance in the statistical analysis [49, 50]. Nineteen studies (41 %) blinded participants to the research question, three studies used blinded outcome assessors (7 %), three studies satisfied both of these criteria (7 %) and 21 studies (46 %) did not state that they used blinding procedures. All of the studies were judged to have used a valid measure of the dependent variable, but only seven studies (15 %) referred to the measure’s reliability. Withdrawal and dropout information was reported in 11 studies (24 %). Concerns relating to intervention integrity were identified in nine studies (20 %).

### Study and Participant Characteristics

Thirty-eight studies (83 %) used group designs, and eight studies (17 %) used single-subject designs. Twenty-nine studies (63 %) were conducted in a laboratory setting, and 17 studies (37 %) were conducted in a field setting. The studies measured running (*n* = 23), cycling (*n* = 14), swimming (*n* = 4), gymnasium triathlon (*n* = 2), rowing (*n* = 2) and walking (*n* = 1) performance, using time trials (alone or in a group, *n* = 25), incremental tests (*n* = 10), constant workload tests (*n* = 6), constant duration tests (*n* = 4) and points won in competition (*n* = 1). Distances in time trials ranged from 1.5 to 20 km in cycling, 1 to 5 km in running and 100 m to 1,000 yards (914 m) in swimming. The number of participants per study ranged from 1 to 90 (mean ± SD = 27 ± 25). The number of males ranged from 0 to 60 (mean ± SD = 17 ± 16), and the number of females ranged from 0 to 45 (mean ± SD = 11 ± 14). Twenty-one studies (46 %) chose a sample of athletes, who ranged in ability from high school to nationally ranked athletes. Two studies [44, 45] chose competitive athletes and measured endurance performance in actual competition. Eleven studies (24 %) considered moderating variables.

### Practical Psychological Interventions

Twenty-five studies measured the effect of a psychological manipulation that was judged to be ethical, feasible and accessible to a practitioner, coach or athlete. Across these 25 studies, 46 manipulations were included. With consideration to potential mediating variables, eight studies (32 %) measured psychological variables, three of these studies [44, 49, 53] explicitly targeted the psychological variable with the intervention and four interventions that improved endurance performance appeared to reduce perception of effort [48, 49, 53, 54]. Eleven studies (44 %) clearly included a social validity or consumer satisfaction measure, and two studies [47, 53] included a placebo control. Three studies referred to the qualifications or the experiences of the person delivering [44, 55] or overseeing [48] the intervention. The interventions were organized into eight categories, and each category is summarized separately. Table S2 in the Electronic Supplementary Material provides additional details on each intervention.

#### Association and Dissociation

Participants were encouraged to use an associative or dissociative cognitive strategy in five studies (one strong quality, two moderate and two weak), and the findings were mixed. The strong-quality study found that a dissociative cognitive strategy increased walking time to exhaustion (Δ = 1.06) in 11 of 14 (79 %) army males [54]. Although the dissociation group performed for 48 % longer than a control group, ratings of perceived exertion were similar in the final minute of performance, which suggests that dissociation reduced the rate of increase in perception of effort. Each of the other four studies compared the effects of multiple interventions. An associative cognitive strategy improved non-athletes’ 1.5-mile running performance to a greater extent than a dissociative strategy, pre-performance psyching up and no intervention [56], but non-athletes who used associative, dissociative or positive self-talk strategies ran similar distances in 30 minutes to those run by a control group [57]. In a single-subject design [58], university rowers who listened to an associative audiotape (Δ = 6.58, PND = 100 %) showed a greater increase in the distance rowed during 40 minutes than those who dissociated by watching a videotape (Δ = 1.63, PND = 92 %) or listening to music (Δ = 0.57, PND = 30 %). Finally, association (Δ = 0.46) and dissociation (Δ = 0.88) improved non-athletes’ 1.5-mile running performances compared with those of participants who were given relaxation exercises as a placebo [47], but baseline running times suggested that the groups were not equivalent. Of the five studies, none included a social validity measure completed by the participants, although a coach reported that rowers who used association developed superior rowing technique [58]. Moderating variables were not considered.

#### Goal Setting

Goal setting improved endurance performance in two studies (combined Δ = 0.34). High-school runners who were assigned easy, challenging and unrealistic combinations of short-term and long-term goals showed similar levels of improvement in their 2.3 km running times (∆ = 0.36) in simulated competition [59]. The amount of improvement was correlated with both ego orientation (*r* = 0.39) and task orientation (*r* = 0.38). A second, moderate-quality study found that non-athletes cycled for longer during an incremental test (Δ = 0.33) when they set themselves a goal for improved endurance performance [51].

#### Hypnosis

Hypnosis interventions improved endurance performance in two studies (one moderate quality and one weak). Hypnotized non-athletes who listened to a motivational passage increased their performance time in an incremental running test, but a control group did not [50]. Participants who demonstrated high hypnotic susceptibility improved their endurance performance (∆ = 0.80), but participants with low susceptibility did not (∆ = 0.13). Additionally, the improvement of high-susceptibility participants was not significantly greater than the improvement of non-hypnotized participants who listened to the same passage. A second study, which used a single-subject design, found that hypnosis led to two of three nationally ranked cyclists winning more points in competitive road races (∆ = 1.85, PND = 52 %). The intervention was designed to increase the intensity of the flow state, and it was delivered by a researcher trained in hypnotic techniques [44].

#### Imagery

One of two studies found that imagery improved endurance performance (both moderate quality). Non-athletes who used pre-performance imagery of skill execution, successful performance outcomes or both showed improvements in 1.5-mile running performance similar to those of a control at the second of two posttests [60]. In a study that used a single-subject design, imagery training and listening to a recording of an imagery script improved three of four competitive youth swimmers’ performances (Δ = 3.32, PND = 75 %) in a 1,000-yard practice set [55]. The imagery training was delivered by a researcher who had experience delivering imagery interventions.

#### Pre-Performance Statements

Four studies (two moderate quality and two weak) found that pre-performance interventions involving instructional or motivational statements improved middle-distance running performance (combined Δ = 0.24). Participation in a group motivational exercise (Δ = 0.10) [61] and motivational and instructional statements delivered by headphones (Δ = 0.09) [46] improved high-school distance runners’ performances in a 1-mile run. These interventions led to greater improvements in endurance performance than yoga exercises and a self-selected song, respectively. In the latter study, however, participants raced in more competitive trials in the posttest, meaning that some improvement in all conditions might be attributable to competition. A third study found that collegiate cross-country runners who listened to statements through headphones significantly improved their 1-mile run performances in three of six intervention conditions (Δ ranged from 0.03 to 0.18) [62]. Finally, motivational statements (Δ = 1.89), instructional statements (Δ = 2.11) and questions about what participants were thinking and feeling (Δ = 1.33), delivered by research assistants, improved six collegiate cross-country runners’ performances in a 1 km run [33]. Each of the four studies assessed participants’ satisfaction with the intervention, one intervention improved endurance performance in simulated competition [46] and one study [46] considered moderating variables. None of these studies measured psychological mediating variables or included multiple posttests. In all four studies, participants chose statements after the baseline performance and only those assigned to certain experimental conditions used all of the statements; this suggests that there could have been subsequent contamination or co-intervention effects.

#### Psychological Skills Training Packages

PST improved endurance performance in five studies (two moderate quality and three weak). First, PST improved the competitive performances of national-level youth swimmers. Across all competitive distances, 23 of 33 participants (70 %) improved their performance following the intervention. Across the five endurance-distance events, however, PST improved group-level endurance performance for those who competed 1 month post-intervention (∆ = 0.28) but it did not improve the performances of those who competed immediately post-intervention (∆ = 0.03) [45]. Second, PST increased the distance run by non-athletes during 90 min in the heat (∆ = 0.54) without increasing ratings of perceived exertion [48]. Furthermore, PST improved the 1,600 m running performances of athletes of varying abilities [39] and non-athletes’ performances in gymnasium triathlons [40, 41]. The interventions improved endurance performance for all 13 participants in single-subject research designs (∆ = 3.70, PND = 84 %), and these improvements were maintained in three to eight posttests [39–41]. PST was somewhat beneficial in actual competition [45], and it improved endurance performance in simulated head-to-head competition [41]. Four studies presented support for the social validity of the intervention [39–41, 45]. Moderating variables were not considered.

#### Relaxation and Biofeedback

A 6-week training programme using relaxation and biofeedback improved the running economy of sub-elite long-distance runners [63]. Peak running velocity was measured to monitor changes in fitness, but it did not change.

#### Self-Talk

Four of five studies supported self-talk interventions (three moderate quality and two weak). Motivational self-talk reduced perception of effort and increased cycling time to exhaustion for 10 of 12 (83 %) non-athletes (∆ = 0.66) [49], and it improved non-athletes’ performances in a 10 km cycling time trial, compared with neutral self-talk (∆ = 0.39) [53]. Non-athletes who used positive self-talk, however, ran similar distances in 30 min to those of participants who used association, dissociation or no strategy [57]. In a single-subject design, positive self-talk statements used with (∆ = 4.56, PND = 100 %) or without (∆ = 2.35, PND = 100 %) audiotape assistance increased the amount of work completed by non-athletes during 20 minutes of cycling [64]. Negative self-talk statements did not improve endurance performance (∆ = 0.48, PND = 37 %). A fifth study found that nationally ranked swimmers swam faster training times when they were using positive thinking (e.g. ‘I’m doing great’), mood words (e.g. ‘blast’) or task-relevant thinking (e.g. ‘elbows up’) than when they were thinking ‘normally’ [65]. As each swimmer was trained in each intervention, however, the control condition might have been contaminated by a strategy that they had learned previously and the swimmers may have used multiple interventions together (i.e. co-intervention). Of the five studies, none considered moderating variables and none included a social validity measure. The four group-design studies included only one posttest.

### Additional Psychological Determinants

Twenty-one studies identified additional psychological factors that affect endurance performance; these psychological determinants were categorized as external motivators (*n* = 10), mental fatigue (*n* = 3), priming interventions (*n* = 3), experimenter effects (*n* = 3), emotion suppression (*n* = 1) and efficacy strength (*n* = 1). With consideration to potential mediating variables, 13 studies measured perception of effort during endurance performance. The findings relating to each psychological determinant are summarized separately.

#### External Motivators

Ten studies (eight moderate quality and two weak) examined the effects of head-to-head competition, verbal encouragement, financial incentives or co-participation on endurance performance. Competition (combined Δ = 0.32) and verbal encouragement (combined Δ = 1.22) generally improved endurance performance. Head-to-head competition improved endurance performance in constant workload tests [31, 66], and it improved time trial performance in one of two studies [67, 68]. On the basis of reported data, 29 of 34 non-athletes (85 %) performed better when competing. Verbal encouragement improved performance in an incremental test in two studies. Specifically, competitive runners (Δ = 2.73) and non-athletes (Δ = 1.58) ran for longer in a verbal encouragement condition [69]. Type B personality non-athletes (Δ = 0.45), but not Type A non-athletes (Δ = 0.03), also ran for longer when encouraged [70]. A financial incentive did not affect regional-level cyclists’ performances in a time trial [71], but a combined intervention of encouragement and incentives improved university endurance athletes’ performances in an incremental running test (Δ = 0.40) [72]. Performing a time trial with another participant (without instructions to compete) did not affect (Δ = −0.07) running performance [73], but performing with a visual representation of another participant did improve (Δ = 0.26) cycling time trial performance [74].

#### Mental Fatigue

Three studies (one strong quality and two moderate) showed that mental fatigue undermines endurance performance. In a strong-quality study, 13 of 16 non-athletes (81 %) demonstrated a reduced time to exhaustion (Δ = 0.34) following a prolonged and demanding cognitive task [75]. The same intervention also led to slower running times (Δ = 0.27) in a 3 km time trial [76]. Furthermore, performing a demanding cognitive task that required response inhibition caused slower performance times in a 5 km running time trial (Δ = 0.34), compared with a similar task without response inhibition [77]. The mentally fatiguing tasks increased perception of effort during subsequent endurance exercise in all three studies.

#### Priming

Three studies examined the effects of priming interventions on endurance performance (all moderate quality). Two experiments examined the effects of subliminally presented visual cues on cycling time to exhaustion [52]. Happy faces reduced perception of effort and led to eight of 13 participants (62 %) performing for longer (Δ = 0.26), compared with sad faces (experiment 1), and action words reduced perception of effort and increased the time to exhaustion, compared with inaction words, in a single-subject experiment (experiment 2). A third study found that rowers who were primed for an autonomy motivation orientation performed faster than control-primed and impersonally primed rowers [78].

#### Experimenter Effects

Three studies (all moderate quality) examined the effects of experimenter characteristics and behaviours on performance in an incremental walking/running test. An experimenter’s sex influenced non-athletes’ endurance performance, depending on the sex and race of participants, but endurance performance was not affected by whether participants and the experimenter were friends [79]. Talking to non-athletes during the test did not affect endurance performance (Δ = −0.10) [80].

#### Emotion Suppression

Instructing endurance athletes to conceal their emotions while watching a disgusting video led to an increase in ratings of perceived exertion and a poorer performance in a subsequent 10 km time trial (Δ = 0.34), compared with watching the video without instructions [81].

#### Efficacy Strength

Youth swimmers with high efficacy strength performed better in simulated competition than those with low efficacy strength [82]. Efficacy strength was manipulated by assigning a goal that was faster (low efficacy strength) or slower (high efficacy strength) than their personal best.

## Discussion

A systematic literature review was conducted to identify psychological determinants of endurance performance. First, this review identified 25 studies that examined the effects of practical psychological interventions on endurance performance. These psychological manipulations were judged to be ethical, feasible and accessible to a sport practitioner, coach or athlete. Twenty-one additional studies were identified that drew attention to other psychological factors that affect endurance performance.

### Practical Psychological Interventions

#### Overview

This review found substantial support for using practical psychological interventions to improve endurance performance. Association, dissociation, goal setting, hypnosis, imagery, pre-performance statements, PST packages and self-talk were found to improve performance in endurance tasks. Of the 24 studies that aimed to improve endurance performance, 22 found that at least one intervention improved performance. With the exception of research conducted on association and dissociation, however, none of the studies compared the effects of these different interventions on endurance performance. For example, PST packages were not compared with their individual components (i.e. goal setting, imagery, relaxation or self-talk), and only one study [57] compared a PST intervention (positive self-talk) with alternative interventions (association and dissociation). Therefore, and because only one study [54] was classed as strong in quality, it is difficult to draw a strong conclusion about whether one practical psychological intervention should be chosen over others. There was, however, consistent support for PST packages, with five studies finding that PST packages improved endurance performance across three sports, with athletes, in real-life and simulated competition, and in multiple posttests [39–41, 45, 48]. The relative contribution of each intervention component is not known [48], however, and support was also found for goal setting, imagery and self-talk interventions in isolation. Therefore, a PST package might be more time consuming for an athlete without further improving their endurance performance. A cautious comparison of effect sizes and PND values does not suggest that there are substantial additive effects, although teaching multiple psychological skills might be advantageous if it allows athletes to choose one or more psychological skills that complement their needs and preferences.

#### Psychological Mediating Variables

Although many practical psychological interventions improved endurance performance, little is known about the psychological mechanisms underlying these improvements. Surprisingly, only three practical psychological intervention studies [44, 49, 53] appeared to target and measure psychological mediating variables. Understanding mediating variables could help sport practitioners and athletes to choose an intervention that might be particularly valuable for that athlete. For example, an intervention that increases self-efficacy could be useful for an athlete who doubts the attainability of their goals during the most demanding periods of a race. Additionally, understanding mediating variables could help a coach or practitioner to adapt the intervention to meet the needs of the athlete, while maintaining the ‘essence’ or intention of the chosen intervention. Measuring mediating variables could also help researchers understand why an intervention was not efficacious for a proportion of participants—that is, the intervention might have had an inconsistent effect on the mediating variable. The findings of this review suggest that practical psychological interventions aimed at increasing motivation, increasing efficacy strength or reducing perception of effort could improve endurance performance. Researchers could therefore design an intervention that targets these psychological factors or examine the effect of an intervention on measures of these psychological factors. Psychological theories could also determine which psychological factors are targeted and measured. As explained in Sect. 4.3, the lack of theoretically informed interventions could account for the paucity of studies investigating psychological mediating variables.

#### Placebo Control Conditions

Increased expectations of performance improvement might account for the effects of some psychological interventions. The placebo effect refers to a favourable outcome that arises purely from a person’s belief that they have received a beneficial treatment [83]. A recent literature review [24] reported placebo effects of varying magnitudes from studies that measured the performance of sub-elite athletes in strength, pain tolerance and endurance tasks ranging from 1 km running to 40 km cycling. Similarly, a recent meta-analysis of 14 studies reported a moderate effect size (0.40) for the placebo effect across exercise modes and performance variables, and a small effect size (0.22) for endurance exercise [25]. In the present review, nine of the 20 effect sizes [45, 46, 61, 62] calculated for practical psychological interventions in group-design studies without a placebo control group were less than 0.22. As well as expecting to improve, participants might have believed that the researchers hoped or expected that they would perform better post-intervention, and they might therefore have offered different amounts of effort in these performance tests (i.e. demand characteristics) [39]. It is difficult to judge the contribution of expectation effects in this review, because only two studies [47, 53] included a placebo control group. Additionally, some of the included studies [39, 50, 54] appeared to heighten participants’ expectations of performance improvement through the wording of the instructions they gave [24]. Furthermore, relatively few studies used research assistants who were blinded to the participants’ allocation, deceived participants about the research question, played down the likely benefits of the intervention (if necessary) or looked at the endurance performance of high-level and motivated athletes in competition; each of these factors might have increased the likelihood of expectation effects in the included studies [83]. It is acknowledged that enhanced expectations can be an important component of a performance enhancement intervention. Nevertheless, it is important for the credibility of sport psychology as a profession that recommended psychological interventions are shown to have greater effects than the expectations they instil in athletes. Unlike other sport science disciplines (e.g. nutrition), psychologists are unable to create a placebo treatment by removing the key ingredients from an intervention. We therefore encourage sport psychologists to compare psychological interventions with alternative control treatments [84] or inert solutions, pills or capsules that are described as beneficial for endurance performance. Alternative control treatments are similar in duration, perceived value and procedure to the experimental treatment, but they target unrelated dependent variables [84]. We also suggest that researchers measure each participant’s expectation of performance improvement [85].

#### Limitations of Practical Psychological Intervention Studies

Additional limitations were consistently identified across the included studies investigating the effects of practical psychological interventions on endurance performance. Only six of the 18 studies that chose group designs reported using random assignment to experimental and control groups, which is an indicator of strong experimental research [86]. None of those six studies, however, measured the endurance performance of athletes in competition. Andersen [87] argued that few randomized, controlled trials have shown that psychological interventions improve the performance of athletes in competition. Further, there are few sport psychology intervention studies that have measured the performance of athletes in competition [35]. Illustrating this point, only two of the included studies [44, 45] examined the effects of an intervention for athletes in real-life competition. These interventions were inconsistent in improving endurance performance, perhaps because of confounding variables (e.g. the specific competition) or because the margins for improvement are small for trained athletes in competition. Alternatively, the benefits of practical psychological interventions for competitive athletes might not be observable in their short-term competitive performances. Instead, psychological strategies that help athletes to improve their performances in training—where performance incentives are likely lower—could lead to meaningful long-term improvements in competitive performances through a physiological mechanism (e.g. adaptation) or a psychological mechanism (e.g. increased self-efficacy). Nevertheless, research that measures endurance performance in competition could complement well-controlled studies by demonstrating whether research findings generalize to ‘real-life’ performance. It is acknowledged that gatekeepers to athletes, such as a coach, might be hesitant to accept that only a proportion of the athletes will receive a potentially beneficial intervention. Researchers and gatekeepers might therefore agree that control participants will be offered the intervention after the study is completed.

The long-term benefits of practical psychological interventions are unclear. For example, none of the studies that delivered instructional or motivational statements before performance included multiple posttests, and the novelty of these and other interventions might wear off. Alternatively, continued practice of a psychological skill could lead to additional improvements in endurance performance. Identified group-design studies did not include more than two posttests, and the second posttest was conducted up to 1 month after the first [45, 60]. Single-subject designs included up to nine post-intervention performances [44], and these studies typically demonstrated that improvements in endurance performance were maintained. However, the effects of practical psychological interventions on endurance performance after three or more months are unknown. It would be valuable to know if participants maintained their improvements in endurance performance, but it would also be difficult to attribute long-term changes in endurance performance to the intervention. Therefore, it would also be valuable to know whether participants continued to use the taught intervention after they finished their commitment to the study [39, 40]. None of the studies reported this information.

When participants were required to make a commitment to a practical psychological intervention, 12 of the studies (67 %) did not report the numbers of withdrawals and dropouts (and the reasons for them). This information is important because participants might drop out when they do not believe that an intervention will be beneficial, when an intervention is not enjoyable or when an intervention is perceived to be inconvenient or too much work. Therefore, dropouts could lead to a greater reported mean improvement in the experimental condition. Furthermore, only three studies referred to the experience or qualifications of the person performing [44, 55] or overseeing [48] the intervention. This information would be valuable so that readers can judge whether the expertise of this person influenced the effects of the intervention. Similarly, practitioners could judge whether they have sufficient expertise to perform the intervention.

### Additional Psychological Determinants

External motivators, mental fatigue, priming, emotion suppression, efficacy strength and the experimenter’s sex affected endurance performance. In particular, experimental research consistently demonstrates that mental fatigue undermines endurance performance, whereas external motivators can have a beneficial effect on endurance performance. Mental fatigue, induced by prolonged and demanding cognitive tasks, consistently increased perception of effort and had a detrimental effect on endurance performance [75–77]. As external motivators, head-to-head competition [31, 66, 67], verbal encouragement [69, 70] and a combined intervention of financial incentives and verbal encouragement [72] improved performance in various endurance tasks, although the introduction of a financial incentive did not affect endurance performance [71]. It is difficult to establish how these interventions improved endurance performance and to explain the inconsistencies in the results, because the effects of the interventions on psychological variables were not measured in these studies. Although head-to-head competition and verbal encouragement might increase participants’ motivation to perform, these interventions might also act as sources of self-efficacy (vicarious experience and verbal persuasion, respectively, e.g. Bandura [88]) or they could reduce perception of effort. Measuring these mediating variables could clarify the psychological mechanisms underlying the observed change in endurance performance. Finally, endurance performance can be affected by priming interventions [52, 78]; additional research is required, however, to determine whether these interventions offer a feasible means of performance enhancement.

### Theoretical Implications

Only three practical psychological interventions [49, 53, 55] tested (or were clearly informed by) a specified psychological theory. Psychological theories can help researchers to identify key factors that determine behaviour (e.g. endurance performance) and that can be targeted by novel or refined interventions [89]—that is, theoretically informed studies could identify the psychological mechanisms through which interventions affect endurance performance, and researchers and practitioners could target these mechanisms with an intervention. Theoretically informed interventions might therefore produce greater or more consistent effects [89].

Although psychological theories have illuminated the effects of psychological factors on perception of effort [90, 91], the psychobiological model of endurance performance is the only model based on psychological theory that specifically explains how psychological factors affect endurance performance. The psychobiological model is based on motivational intensity theory [92], and it argues that perception of effort and potential motivation are the ultimate determinants of endurance performance (for an overview of the psychobiological model, see Smirmaul et al. [93]). Perception of effort is the conscious sensation of how hard, heavy and strenuous the exercise is [94], and potential motivation refers to the greatest amount of effort that a person would be willing to offer to satisfy a motive [95]. The psychobiological model predicts that any psychological or physiological factor that increases potential motivation or reduces perception of effort will improve endurance performance, and that any psychological or physiological factor that reduces potential motivation or increases perception of effort will undermine endurance performance [96]. Similarly, the psychobiological model suggests that all psychological and physiological manipulations that affect endurance performance do so because they affect either potential motivation or perception of effort [97]. In support of the psychobiological model, motivational self-talk [49, 53], PST [48] and dissociation [54] reduced perception of effort and improved endurance performance; mentally fatiguing tasks [75–77] and emotion suppression [81] increased perception of effort and undermined endurance performance; and subliminally presented visual cues [52] influenced both perception of effort and endurance performance. Future research that includes psychological mediating variables (e.g. perception of effort) could clarify the psychological mechanisms through which psychological manipulations affect endurance performance. Although the psychobiological model emphasizes perception of effort and potential motivation, researchers are encouraged to include additional psychological mediating variables, such as self-efficacy, that could shed light on the psychological mechanisms underlying changes in endurance performance.

In the present review, five studies explicitly examined the effect of traditional associative and dissociative attentional strategies on endurance performance [47, 54, 56–58]. Traditional classifications of attentional focus have proposed that athletes who use an associative strategy monitor their bodily sensations and use this feedback to adjust their pace; athletes who dissociate direct their attention away from these uncomfortable sensations [98]. More recent theoretical perspectives, however, argue that attentional strategies can be categorized more precisely [9, 99]. Brick and colleagues [9] recently proposed a five-category model of attentional activity. According to this model, athletes ‘actively self-regulate’ when they attempt to control or monitor their thoughts, feelings or actions. This associative strategy allows an athlete to optimize their pace or efficiency of movement without elevating perception of effort. Examples include self-talk and relaxation strategies that are used during endurance performance [41] and pre-performance; mental preparation strategies, such as setting process goals [46]; and visualizing successful execution of skills [55]. The findings of the present review suggest that active self-regulation strategies could be valuable for athletes who aim to optimize their endurance performance.

### Implications and Recommendations for Practice

PST interventions involving imagery, self-talk and goal setting offer a promising tool for improving the performance of endurance athletes. It is unclear whether teaching multiple psychological skills is more beneficial than teaching one psychological skill, particularly when the intervention is tailored to meet the needs of an athlete. As mental fatigue increases perception of effort and undermines endurance performance, endurance athletes should avoid mentally draining activities before they compete. For example, endurance athletes might avoid thought suppression strategies (e.g. thought stopping) and situations that require them to suppress their emotions or behaviour (e.g. interviews with the press) before they compete, because they could be mentally fatiguing [81, 100] and therefore detrimental to endurance performance. As a further suggestion, coaches could use head-to-head competition and verbal encouragement during training to facilitate maximum effort when required (e.g. sprint interval training).

Music could be valuable during training, as well as events and competitions that permit its use. Although excluded from this review, there is substantial evidence that music elicits positive affect and feeling states during exercise at all intensities, reduces perception of effort during exercise below the lactate threshold and facilitates endurance performance [27, 28]. Self-selecting music for its motivational qualities is encouraged [27, 28]. The benefits of music, however, should be weighed against potential risks, such as not hearing safety-related cues (e.g. road traffic), distraction from technique or distraction from pacing-related cues (e.g. bodily sensations and other competitors). Placebos and various forms of deceptive feedback can also be used to improve endurance performance; the practical application of these manipulations during training and competitions, however, raises significant ethical issues [26, 101].

### Implications and Recommendations for Research

Theoretically driven studies could systematically examine the mechanisms through which psychological interventions affect endurance performance, and they could therefore encourage development and refinement of performance enhancement interventions that have consistent and strong effects in endurance events. Research examining the effects of interventions in real-life competition could particularly add to the endurance literature. Researchers are also encouraged to compare different performance enhancement interventions, using randomized, controlled experimental designs. Inclusion of placebo control conditions could help readers to judge the effects of interventions beyond expectation effects. Furthermore, these studies should include more than one posttest, report whether participants continue to use the intervention following their commitment to the study, report the number of participants who drop out from the study and their reasons for doing so, and provide expertise-related information on the person delivering the intervention.

As an alternative to measuring performance in real-life endurance competition, researchers could use head-to-head competition and verbal encouragement to ensure that participants offer maximum effort during an endurance task. This could help researchers to test the effects of interventions when participants are in motivated performance situations. To reduce the risk of confounding variables, care should be taken to apply head-to-head competition and verbal encouragement consistently across experiment trials. For example, a research assistant who is blinded to the study aims or hypotheses could provide verbal encouragement using a consistent verbal encouragement protocol [102], a blinded and independent researcher could analyse audio recordings of the delivered verbal encouragement and attempt to predict the experimental conditions, and head-to-head competition procedures could be standardized [67].

Few practical psychological interventions appeared to be designed specifically for the demands of endurance sports. More often, interventions were informed by research on mental preparation or interventions across a range of sports. This is surprising because endurance activities have physical, technical, logistical and psychological demands that should be taken into account when an intervention is being designed [103]. Qualitative research has drawn some attention to the demands faced by endurance athletes and the cognitive strategies used by high-level endurance athletes [104–108]. Future research could shed greater light on the demands facing endurance athletes or test interventions that are designed to help athletes to cope with these demands.

It is surprising that only four studies [75–77, 81] examined interventions that undermine endurance performance. Ethically approved future research could address the effects of other psychological states (e.g. debilitative anxiety), psychological strategies (e.g. thought suppression) and situations that athletes encounter (e.g. insufficient time for mental preparation) that could be debilitative to performance, in experimental endurance tasks.

Little is known about whether participant characteristics influence the effects of psychological manipulations. The results shed little light on whether sex [46, 60, 79, 80] or athletic ability [69, 82] are moderating variables. Nevertheless, personality type appears to affect participants’ responses to verbal encouragement [70], participants with high task and ego orientations respond more favourably to goal-setting interventions [59], and hypnotic susceptibility influences whether hypnosis-based interventions improve endurance performance [50]. Further research on moderating variables (e.g. competitive level, competitive distance, achievement-goal orientation) could shed light on whether certain interventions are particularly beneficial for specific groups of athletes, and this evidence base could increase the effects of sport psychology interventions.

Lack of blinding procedures was often a source of bias. Researchers who are aware of the intervention status of participants might unintentionally affect participants’ performance expectations. Where resources are available, researchers are encouraged to collect data using research assistants who are blinded to treatment allocation, particularly when verbal encouragement is given during endurance performance. It is acknowledged that researchers may be unable to disguise the research question when they are testing the effects of an intervention. Researchers could therefore inform participants that they do not know what impact (if any) the intervention will have on their endurance performance [55], or they could include an alternative control treatment [84].

Finally, researchers could consider a more diverse range of sports and distances. None of the located studies examined rowing or triathlon performance in field settings, and none of the research was focused on endurance-distance race walking, speed skating or cross-country skiing. There is also a lack of studies examining the effects of interventions in long-distance events (e.g. half marathons, open-water swims, ultra-distance events); only two studies [44, 48] measured performance in endurance tasks that took longer than 1 h to complete.

### Limitations of the Systematic Review

This literature review synthesized studies on the psychological determinants of endurance performance. A heterogeneous selection of studies was included, and there are insufficient studies to provide sport- or distance-specific guidance. Outcome measures that range from 100 m breaststroke swimming [45] to ultra-endurance events (e.g. long-distance triathlons) could satisfy our definition of endurance performance. The technical, physical, logistical and mental demands [103] of the included sports and distances will undoubtedly vary, and the comparability of these performance measures could therefore be questioned. Individual differences also need to be taken into account; interventions seemed influential for only a proportion of group-design participants. While the findings of this systematic review should inform evidence-based practice, practitioners interested in performance enhancement should also consider the demands of the specific sport and competitive distance, as well as the needs of the individual athlete [103].

This systematic review synthesized the peer-reviewed studies that have been published to date, because these studies comprise the evidence base that is available to practitioners, theorists and researchers. Publication bias might partially account for the abundance of interventions that significantly affected endurance performance, because studies might not have been put forward or accepted for publication if the examined intervention did not have an effect [34]. Indeed, a recent study reported statistical evidence that publication bias is a pervasive problem across all areas of psychological research [109].

Each included study was evaluated using a modified version of the EPHPP Quality Assessment Tool for Quantitative Studies [32]. This evaluation tool is not specific to the sport context, and it was therefore adapted. The tool evaluates information that is reported in the manuscript, and reporting practices could vary between healthcare and sport science. Nevertheless, researchers are encouraged to report randomization and blinding procedures (when performed) and the numbers of withdrawals and dropouts (and the reasons for them), because this information is important for judging bias. An evaluation tool that is specific to sport science research and is sensitive to its research practices would be valuable. Similarly, an evaluation tool that recognizes the strengths of single-subject research in sport psychology (see Barker et al. [110]), as well as the different quality criteria applied to these designs, would be welcomed. The tool was useful, however, for identifying common sources of bias across all of the studies, such as blinding and withdrawals and dropouts, and comparing the quality of the included studies.

## Conclusion

This systematic literature review aimed to identify psychological determinants of endurance performance. Additional objectives were to evaluate the research practices of included studies, to suggest theoretical and applied implications, and to guide future research. Of the practical psychological interventions identified, consistent support was found for using imagery, self-talk and goal setting to improve endurance performance, but it is unclear whether learning multiple psychological skills is more beneficial than learning one psychological skill. The results also demonstrated that mental fatigue undermines endurance performance, and verbal encouragement and head-to-head competition can have a beneficial effect. Consistent with the psychobiological model of endurance performance, interventions that influenced perception of effort consistently affected endurance performance. Researchers are encouraged to compare different practical psychological interventions, to examine the effects of these interventions for athletes in competition and to include a placebo control condition or an alternative control treatment. Researchers are also encouraged to explore additional psychological factors that could have a negative effect on endurance performance. Future research should include psychological mediating variables and moderating variables.

## Electronic supplementary material

Below is the link to the electronic supplementary material.

Supplementary material 1 (DOCX 20.1 kb)

Supplementary material 2 (DOCX 53.3 kb)

Supplementary material 3 (DOCX 78.7 kb)

Supplementary material 4 (DOCX 16.6 kb)
