# Supplementary material for: Psychological Determinants of Whole-Body Endurance Performance
Source: Sports Med. 2015 Mar 15;45(7):997–1015. doi: 10.1007/s40279-015-0319-6 (PMC4473096; doi:10.1007/s40279-015-0319-6)
Supplement: Supplementary file 1 — Supplementary material 1 (DOCX 20.1 kb) [file 40279_2015_319_MOESM1_ESM.docx]

Psychological Determinants of Whole-Body Endurance Performance

*Sports Medicine*

Alister McCormick (🖂), Carla Meijen, and Samuele Marcora

Endurance Research Group, University of Kent
E-mail: [am801@kent.ac.uk](mailto:am801@kent.ac.uk)

Electronic Supplementary Material Appendix S2. Modified Effective Public Health Practice Project (EPHPP) application script.

Modified Effective Public Health Practice Project (EPHPP) Application Script

Studies were evaluated against their own research aims. “Not applicable” was assigned when an evaluation criterion was judged to not be relevant to the aims of a study. Judgments were made when the “correct” rating was unclear.

**Selection Bias**

The EPHPP dictionary explains that participants are very likely to be representative of a target population if they are randomly selected from a comprehensive list of individuals in that target population (“strong” rating). Participants are somewhat likely to be representative of a population (“moderate”) if they are systematically referred from a source (e.g., clinic, coach). Participants are unlikely to be representative if they are self-referred (“weak”).

**Study Design**

The EPHPP dictionary explains that, “In stronger designs, an equivalent control group is present and the allocation process is such that the investigators are unable to predict the sequence” and that, in an experimental study, “raters assess the likelihood of bias due to the allocation process”.

Strong ratings:

- Between-subjects and pretest-posttest group designs that included control groups and randomly allocated participants to groups (with or without matching procedures).
- Within-subject designs where the order of experimental and control conditions was completely randomised (or randomised and counterbalanced).

Moderate ratings:

- Between-subjects and pretest-posttest group designs that included control groups but did not state using randomisation.
- Within-subject group designs with counterbalancing but not stating randomisation.
- Quasi-experimental group designs (e.g., randomisation of pre-existing groups, allocation by participant characteristics).
- Pretest-posttest group designs with multiple pretests and no control group.

Weak ratings:

- Single-subject, multiple-baseline designs.
- Pretest-posttest group designs without a control group and only one pretest.
- Studies where the design could not be determined. In contrast to the EPHPP, attempts were made to determine the research design if it was not explicitly stated (rather than assigning “weak”).

**Confounders**

The EPHPP dictionary defines a confounder as, “a variable that is associated with the intervention or exposure and causally related to the outcome of interest”. In line with the EPHPP dictionary, attempts were made to determine whether groups were balanced with respect to important variables prior to the intervention, and if attempts were made to control confounding variables in the design (e.g., through matching procedures) or analysis (e.g., through appropriate use of statistics). When designs included more than one experimental group or a control group, the pre-intervention performances of the groups were compared. Other variables that may also affect performance (e.g., age, gender, competitive level, competition) were also compared. An attempt was made to identify potential confounders throughout the description of the research method. The reviewer considered whether there were any differences (e.g., environmental, procedural) between experimental and control conditions, other than the intervention itself. Within-subject designs were often classed as “strong”, because participants acted as their own control in consistent environments.

In contrast to the EPHPP, “weak” was not assigned if the control of confounders was not described, because it is not typical practice to report this information in sport science research.

**Blinding**

Judgments were made regarding whether the expectations of the researcher or the participants could have affected the outcome measure. First, a judgment was made regarding whether researchers who were present at post-intervention testing were blinded to the intervention-status of participants (i.e., whether participants were in an experimental or control condition) or the research aims or hypotheses. The reviewer looked for reference to a blinded research assistant. Second, a judgment was made regarding whether the participants were aware of the research question. The reviewer considered whether studies used deception or concealment of information and whether they informed participants of the likely effects of the intervention on performance. The reviewer also considered whether the research question was likely to be obvious to participants (e.g., the effect of an intervention on performance). As few articles explicitly described blinding, a judgment was made using the information available in the article. “Strong” was assigned when both the researcher and the participant were blinded. “Moderate” was assigned when either the researcher or the participant were blinded, or when blinding was unclear in both instances. “Weak” was assigned when neither the researcher nor the participant appeared to be blinded.

**Data Collection Methods**

The EPHPP dictionary explains that, “Tools for primary outcome measures must be described as reliable and valid” and that, “Reliability and validity can be reported in the study or in a separate study”. The EPHPP recognises that some standard assessment tools have known reliability and validity. In most cases, the primary data collection tool was a measure of endurance performance. Only studies that were judged to have used a valid measure of endurance performance were included in the systematic review and so, for consistency, all studies that used an endurance-performance measure as the primary dependent variable were judged to have used a valid outcome measure. For those studies that used running economy or V̇O_2max_ as the primary dependent variable, measurement procedures with known validity were considered acceptable. For an outcome measure to be considered reliable, the study was required to quote reliability data, reliability criteria, or a reference supporting reliability.

**Withdrawals and Dropouts**

Reporting withdrawals and dropouts was considered to be relevant for those practical psychological intervention studies that looked at the effect of training in a performance-enhancement intervention on endurance performance (i.e., a considerable commitment of time and effort to the intervention) and practical psychological intervention studies that included more than one posttest / performance in an experimental condition. Participants who dropped out may have been less likely to show a beneficial response to the intervention, and excluding these participants from the analysis could lead to biased conclusions.

**Intervention Integrity**

The reviewer noted whether the consistency of the intervention was measured or whether the intervention was delivered using a standardised procedure (i.e., whether the intervention was provided to all participants in the same way). Additionally, the reviewer noted whether participants were likely to have received an unintended intervention (contamination or co-intervention) that could have influenced the results. Specifically, the reviewer considered whether the experimental group could have received an additional intervention (co-intervention) or whether the control group could have accidentally received the experimental intervention (contamination). For example, to prevent contamination, some studies reported asking participants to not discuss the intervention with other participants.

**Analyses**

None of the studies reported using an intention-to-treat analysis. The reviewer noted the analysis conducted on performance data, as well as whether the authors justified the choice of statistical analysis.
