# Supplementary material for: Psychological Determinants of Whole-Body Endurance Performance
Source: Sports Med. 2015 Mar 15;45(7):997–1015. doi: 10.1007/s40279-015-0319-6 (PMC4473096; doi:10.1007/s40279-015-0319-6)
Supplement: Supplementary file 2 — Supplementary material 2 (DOCX 53.3 kb) [file 40279_2015_319_MOESM2_ESM.docx]

Psychological Determinants of Whole-Body Endurance Performance

*Sports Medicine*

Alister McCormick (🖂), Carla Meijen, and Samuele Marcora

Endurance Research Group, University of Kent
E-mail: [am801@kent.ac.uk](mailto:am801@kent.ac.uk)

| **Electronic Supplementary Material Table S1**. Reasons for study exclusion | | | | |
| --- | --- | --- | --- | --- |
| Authors | Abstract details | | Manuscript details | Reason for exclusion |
| Andreacci et al. [1] | Appears relevant. | | The exercise test ended when the participant either attained V̇O_2max_ or reached exhaustion (p.347). | Dependent variable does not meet inclusion criteria |
| Andreacci et al. [2] | Does not appear relevant from the abstract (no reference to performance time). The manuscript was studied, however, because endurance time was likely to have been measured. | | The criteria for terminating the exercise test were unclear, so I contacted the author. The author stated, "Exercise time was the total time that a person spent on the treadmill and ended when they terminated the test or met V̇O_2max_ criteria." | Dependent variable does not meet inclusion criteria |
| Anshel [3] | Appears relevant. Two endurance durations. | | Performance was not measured in consistent experimental conditions (individual performance versus simulation of a rowing team). | Performance was not measured in consistent experimental conditions. |
| Baghurst et al. [4] | Appears relevant. | | No control group. | No control condition. |
| Bandura and Cervone [5] | Relevance unclear. | | The "stenuous activity" (pulling and pushing two arm levers) does not meet the endurance definition of the review. | Dependent variable does not meet inclusion criteria |
| Bar-Eli and Blumenstein [6] | Relevance unclear. | | Swimming and running distances do not meet the inclusion criteria (all ˂ 35 s). | Dependent variable does not meet inclusion criteria |
| Bar-Eli and Blumenstein [7] | Relevance unclear. | | Performance distances (30 m) do not meet the inclusion criteria. | Dependent variable does not meet inclusion criteria |
| Bar-Eli et al. [8] | Relevance unclear. | | Performance times do not meet the inclusion criteria (all ˂ 40 s). | Dependent variable does not meet inclusion criteria |
| Barling and Bresgi [9] | Unlikely to meet inclusion criteria. | | Performance times are less than 75 s. | Dependent variable does not meet inclusion criteria |
| Beauchamp et al. [10] | Unlikely to be an appropriate design or measure performance objectively. | | Not an experiment. | Design does not meet the inclusion criteria. |
|  |  | |  |  |
| **Table S1** continued | | | | |
| Authors | Abstract details | | Manuscript details | Reason for exclusion |
| Bueno et al. [11] | Did not appear to meet inclusion criteria. | | Confirmation that study does not meet inclusion criteria. This is a mechanism study rather than an applied intervention study. | Design does not meet the inclusion criteria. |
| Burton [12] | Relevance unclear. | | Endurance and non-endurance distances but results not presented separately. | Dependent variable does not meet inclusion criteria. |
| Callow et al. [13] | Appears relevant, depending on the performance distance. | | Performance times < 40 s | Dependent variable does not meet inclusion criteria. |
| Callow et al. [14] | Experiments 2 and 3 appear relevant, depending on the performance distances. | | Experiment 2 - Performance times < 20 s  Experiment 3 - Performance times < 25 s | Dependent variable does not meet inclusion criteria. |
| Carnes et al. [15] | Appears relevant, and distance covered is measured. | | “This is not a test to see how fast or far you can go.” (p.2) | Not full-effort performance. |
| Clingman and Hilliard [16] | Relevance unclear. | | Race-walking performance but not race-walking performance distance (only 0.5 miles, as stated in the abstract) | Dependent variable does not meet inclusion criteria. |
| Connolly and Janelle [17] | Appears relevant. Unclear if there is a control group in Experiment 1. | | Experiment 1 - Association and dissociation but no control. Also not full-effort performance.  Experiment 2 - Control group but not full-effort performance. | Not full-effort performance. |
| Couture [18] | Appears relevant. | | Participants swam as fast as comfortably possible. Therefore not full-effort performance. | Not full-effort performance. |
| De Petrillo et al. [19] | Appears relevant. | | Running performance was not objectively measured. | Dependent variable does not meet inclusion criteria. |
| Diaz-Ocejo et al. [20] | Relevance unclear. Not likely to meet experimental design inclusion criteria. | | Case study. Not an experiment or quasi-experiment. | Design does not meet the inclusion criteria. |
| Everett et al. [21] | Performance distance / duration not stated. | | Performance times are below 75 s. | Dependent variable does not meet inclusion criteria. |
| **Table S1** continued | | | | |
| Authors | | Abstract details | Manuscript details | Reason for exclusion |
| Fillingim and Fine [22] | | Effect of attentional focus on performance. Appears relevant. | Participants were told to “jog as fast as you can without experiencing any discomfort” (p.117). Therefore not full-effort performance. | Not full-effort performance. |
| Fillingim et al. [23] | | Appears relevant. | Not full effort performance. | Not full-effort performance. |
| Goudas et al. [24] | | Appears relevant as measures the effect of goal setting on endurance performance. | Performance was measured by the time point at which the participant reached 170 beats per minute. | Dependent variable does not meet inclusion criteria. |
| Gould et al. [25] | | Abstract not found. | Correlational | Design does not meet the inclusion criteria. |
| Gravel et al. [26] | | Appears relevant. | Performance was not objectively measured and then presented. | Dependent variable does not meet inclusion criteria. |
| Greenlees et al. [27] | | Appears relevant. | 700 m cycling performance < 75 s | Dependent variable does not meet inclusion criteria. |
| Gregg et al. [28] | | Relevance unclear. | The multiple-baseline design did not apply to the performance variable. | Design does not meet the inclusion criteria. |
| Hall and Byrne [29] | | “Endurance task” | Sit up endurance | Dependent variable does not meet inclusion criteria. |
| Hanton and Jones [30] | | Appears relevant. | Performance times are below 75 s. | Dependent variable does not meet inclusion criteria. |
| Hatzigeordiadis et al. [31] | | Appears relevant. Competitive distance not reported. | Endurance and non-endurance distances but results not presented separately. | Dependent variable does not meet inclusion criteria. |
| Kavanagh and Hausfeld [32] | | Relevance unclear as task not specified | Muscular endurance | Dependent variable does not meet inclusion criteria. |
| Kleine et al. [33] | | Appears relevant. | Experiment 1 - The dependent variable is not a measure of endurance.  Experiment 2 - Performance distance from 100 m to 10,000 m presented together. | Dependent variable does not meet inclusion criteria. |
|  | |  |  |  |
| **Table S1** continued | | | | |
| Authors | | Abstract details | Manuscript details | Reason for exclusion |
| LaCaille et al. [34] | | Performance time was a dependent variable. Cognitive strategy was an independent variable. Unclear if there is a control group. | No control condition. Also, it is unclear if participants were required to offer full effort. | No control condition. |
| Padgett and Hill [35] | | Performance time was a dependent variable in Experiment 2. Unclear what “normal training pace” means. Exercise setting was used to manipulate attentional focus so could be relevant. | Still unclear. Email correspondence with the author suggested that it was not full-effort performance. | Not full-effort performance. |
| Pavlidou and Doganis [36] | | Appears relevant. | 50 m performance. | Dependent variable does not meet inclusion criteria. |
| Pennebaker and Lightner [37] | | The abstract provided insufficient information to make a judgment about Experiment 2. | Participants were “instructed that they should jog at whatever pace was comfortable to them, that speed was not an object in the experiment, and that they could walk rather than jog if they so desired” (p.170) | Not full-effort performance. |
| Perreault et al. [38] | | Appears relevant. | Measured changes in % peak power across four stages of performance. Values are reported for 11 of 12 performance minutes. | Dependent variable does not meet inclusion criteria. |
| Rushall et al. [39] | | Appears relevant. | Not considered cross-country skiing performance. The duration of each effort took from 70 to 130 s. | Dependent variable does not meet inclusion criteria. |
| Schomer [40] | | Unlikely to be relevant. This paper is frequently cited, however, and there have been suggestions in the literature that the intervention improved performance. | Performance was not formally / objectively measured. | Dependent variable does not meet inclusion criteria. |
| Schuler and Langens [41] | | Study 2 appears relevant. | Hierarchical analysis of regression | Design does not meet the inclusion criteria. |
| Sewell [42] | | Appears relevant. | Participants performed at 75% of race pace. | Not full-effort performance. |
| Smith et al. [43] | | Relevance unclear. | Sit up endurance | Dependent variable does not meet inclusion criteria. |
| **Table S1** continued | | | | |
| Authors | | Abstract details | Manuscript details | Reason for exclusion |
| Sorrentino and Sheppard [44] | | Appears relevant. The performance duration is likely > than 75 s. | Performance was not measured in consistent experimental conditions (representing theirself versus representing their group). | Performance was not measured in consistent experimental conditions. |
| Spink and Longhurst [45] | | Appears relevant. | “The original group assignments were disregarded and subjects were regrouped according to the actual cognitive strategy employed” (p.11) | Study design does not meet the inclusion criteria. |
| Theodorakis [46] | | Appears relevant. | Performance time = 20 s | Dependent variable does not meet inclusion criteria. |
| Thompson et al. [47] | | Unlikely to meet inclusion criteria because it is a follow-up of an earlier study (De Petrillo et al. [19]) that did not meet the inclusion criteria. | Not an experiment and performance was not measured objectively. | Design does not meet the inclusion criteria. |
| Wanlin et al. [48] | | Appears relevant. | Performance times were below 75 s. | Dependent variable does not meet inclusion criteria. |
| Weinberg et al. [49] | | This is a reaction to Hall and Byrne [29], which was not included. No reference to type of task. | Sit up endurance. | Dependent variable does not meet inclusion criteria. |
| Weinberg et al. [50] | | No abstract. “Motor tasks” | Not endurance. | Dependent variable does not meet inclusion criteria. |
| Williams et al. [51] | | Performance distance / duration not stated. | Performance times are below 75 s. | Dependent variable does not meet inclusion criteria. |
| Wrisberg et al. [52] | | Appears relevant. Unclear if there is a control group. | No control condition. Participants were also described as “normally inactive” in the Introduction. | No control condition. |

Note: Other studies that appear relevant were excluded if there was sufficient information in the abstract to exclude them. For example, the title might include "endurance", but the abstract could clearly state that a muscular endurance task was chosen.

**References**

1. Andreacci JL, Lemura LM, Cohen SL, et al. The effects of frequency of encouragement on performance during maximal exercise testing. J Sports Sci. 2002;20(4):345-52. doi:10.1080/026404102753576125

2. Andreacci JL, Robertson RJ, Goss FL, et al. Frequency of verbal encouragement effects sub-maximal exertional perceptions during exercise testing with young adult women. Int J Sport Psychol. 2004;35(4):267–83.

3. Anshel MH. Examining social loafing among elite female rowers as a function of task duration and mood. J Sport Behav. 1995;18(1):39–49.

4. Baghurst T, Thierry G, Holder T. Evidence for a relationship between attentional styles and effective cognitive strategies during performance. Athl Insight. 2004;6(1):36–51.

5. Bandura A, Cervone D. Self-evaluative and self-efficacy mechanisms governing the motivational effects of goal systems. J Pers Soc Psychol. 1983;45(5):1017-28. doi:10.1037/0022-3514.45.5.1017

6. Bar-Eli M, Blumenstein B. Performance enhancement in swimming: the effect of mental training with biofeedback. J Sci Med Sport. 2004;7(4):454-64. doi:10.1016/S1440-2440(04)80264-0

7. Bar-Eli M, Blumenstein B. The effect of extra-curricular mental training with biofeedback on short running performance of adolescent physical education pupils. Eur Phys Educ Rev. 2004;10(2):123-34. doi:10.1177/1356336X04044055

8. Bar-Eli M, Dreshman R, Blumenstein B, et al. The effect of mental training with biofeedback on the performance of young swimmers. Appl Psychol Int Rev. 2002;51(4):567-81. doi:10.1111/1464-0597.00108

9. Barling J, Bresgi I. Cognitive factors in athletic (swimming) performance: A re-examination. J Gen Psychol. 1982;107(2):227-31. doi:10.1080/00221309.1982.9709930

10. Beauchamp MK, Harvey RH, Beauchamp PH. An integrated biofeedback and psychological skills training program for Canada’s Olympic short-track speedskating team. J Clin Sport Psychol. 2012;6(1):67–84.

11. Bueno J, Weinberg RS, Fernández-Castro J, et al. Emotional and motivational mechanisms mediating the influence of goal setting on endurance athletes’ performance. Psychol Sport Exerc. 2008;9(6):786-99. doi:10.1016/j.psychsport.2007.11.003

12. Burton D. Winning isn’t everything: Examining the impact of performance goals on collegiate swimmers' cognitions and performance. Sport Psychol. 1989;3(2):105–32.

13. Callow N, Roberts R, Fawkes JZ. Effects of dynamic and static imagery on vividness of imagery, skiing performance, and confidence. J Imag Res Sport Phys Act. 2006;1(1):2. doi:10.2202/1932-0191.1001

14. Callow N, Roberts R, Hardy L, et al. Performance improvements from imagery: evidence that internal visual imagery is superior to external visual imagery for slalom performance. Front Hum Neurosci. 2013;7:697. doi:10.3389/fnhum.2013.00697

15. Carnes AJ, Barkley JE, Williamson M, et al. The presence of a familiar peer does not affect intensity or enjoyment during treadmill exercise in male distance runners or non-runners. J Athl Enhanc. 2013;2(4). doi:10.4172/2324-9080.1000119

16. Clingman JM, Hilliard DV. Race walkers quicken their pace by tuning in, not stepping out. Sport Psychol. 1990;4(1):25–32.

17. Connolly CT, Janelle CM. Attentional strategies in rowing: Performance, perceived exertion, and gender considerations. J Appl Sport Psychol. 2003;15(3):195-212. doi:10.1080/10413200305387

18. Couture RT, Jerome W, Tihanyi J. Can associative and dissociative strategies affect the swimming performance of recreational swimmers? Sport Psychol. 1999;13(3):334–43.

19. De Petrillo LA, Kaufman KA, Glass CR, et al. Mindfulness for long-distance runners: An open trial using Mindful Sport Performance Enhancement (MSPE). J Clin Sport Psychol. 2009;3(4):357–76.

20. Díaz-Ocejo J, Kuitunnen S, Mora-Mérida JA. An intervention to enhance the performance of a 3000 metre steeplechase athlete with the use of segmentation and self-talk. Rev Psicol Deporte. 2013;22(1):87–92.

21. Everett JJ, Smith RE, Williams KD. Effects of team cohesion and identifiability on social loafing in relay swimming performance. Int J Sport Psychol. 1992;23(4):311–24.

22. Fillingim RB, Fine MA. The effects of internal versus external information processing on symptom perception in an exercise setting. Health Psychol. 1986;5(2):115–23.

23. Fillingim RB, Roth DL, Haley WE. The effects of distraction on the perception of exercise-induced symptoms. J Psychosom Res. 1989;33(2):241–8.

24. Goudas M, Theodorakis Y, Laparidis K. The effect of external versus internal types of feedback and goal setting on endurance performance. Athl Insight. 2007;9(3):57–66.

25. Gould D, Tuffey S, Hardy L, et al. Multidimensional state anxiety and middle distance running performance: An exploratory examination of Hanin’s (1980) zones of optimal functioning hypothesis. J Appl Sport Psychol. 1993;5(1):85-94. doi:10.1080/10413209308411307

26. Gravel R, Lemieux G, Ladouceur R. Effectiveness of a cognitive behavioral treatment package for cross-country ski racers. Cogn Ther Res. 1980;4(1):83-9. doi:10.1007/BF01173357

27. Greenlees IA, Graydon JK, Maynard IW. The impact of collective efficacy beliefs on effort and persistence in a group task. J Sports Sci. 1999;17(2):151-8. doi:10.1080/026404199366253

28. Gregg MJ, Hrycaiko D, Mactavish JB, et al. A mental skills package for Special Olympics Athletes: A preliminary study. Adapt Phys Act Q. 2004;21(1):4–18.

29. Hall HK, Byrne ATJ. Goal setting in sport: Clarifying recent anomalies. J Sport Exerc Psychol. 1988;10(2):184–98.

30. Hanton S, Jones G. The effects of a multimodal intervention program on performers: II. Training the butterflies to fly in formation. Sport Psychol. 1999;13(1):22–41.

31. Hatzigeorgiadis A, Galanis E, Zourbanos N, et al. Self-talk and competitive sport performance. J Appl Sport Psychol. 2014;26(1):82-95. doi:10.1080/10413200.2013.790095

32. Kavanagh D, Hausfeld S. Physical performance and self-efficacy under happy and sad moods. J Sport Exerc Psychol. 1986;8(2):112–23.

33. Kleine D, Sampedro RM, Melo SL. Anxiety and performance in runners: Effects of stress and anxiety on physical performance. Anxiety Res. 1988;1(3):235–46. doi:10.1080/08917778808248722

34. LaCaille RA, Masters KS, Heath EM. Effects of cognitive strategy and exercise setting on running performance, perceived exertion, affect, and satisfaction. Psychol Sport Exerc. 2004;5(4):461-76. doi:10.1016/S1469-0292(03)00039-6

35. Padgett VR, Hill AK. Maximizing athletic performance in endurance events: A comparison of cognitive strategies. J Appl Soc Psychol. 1989;19(4):331-40. doi:10.1111/j.1559-1816.1989.tb00058.x

36. Pavlidou M, Doganis G. The effects of a psychological intervention program in swimming. J Excell. 2008;12:71–7.

37. Pennebaker JW, Lightner JM. Competition of internal and external information in an exercise setting. J Pers Soc Psychol. 1980;39(1):165-74. doi:10.1037//0022-3514.39.1.165

38. Perreault S, Vallerand RJ, Montgomery D, et al. Coming from behind: On the effect of psychological momentum on sport performance. J Sport Exerc Psychol. 1998;20(4):421–36.

39. Rushall BS, Hall M, Roux L, et al. Effects of three types of thought content instructions on skiing performance. Sport Psychol. 1988;2(4):283–97.

40. Schomer HH. Mental strategy training programme for marathon runners. Int J Sport Psychol. 1987;18(2):133–51.

41. Schüler J, Langens TA. Psychological crisis in a marathon and the buffering effects of self-verbalizations. J Appl Soc Psychol. 2007;37(10):2319-44. doi:10.1111/j.1559-1816.2007.00260.x

42. Sewell DF. Attention-focusing instructions and training times in competitive youth swimmers. Percept Mot Skills. 1996;83(3):915-20. doi:10.2466/pms.1996.83.3.915

43. Smith JA, Hauenstein NMA, Buchanan LB. Goal setting and exercise performance. Hum Perform. 1996;9(2):141-54. doi:10.1207/s15327043hup0902_3

44. Sorrentino RM, Sheppard BH. Effects of affiliation-related motives on swimmers in individual versus group competition: A field experiment. J Pers Soc Psychol. 1978;36(7):704-14. doi:10.1037//0022-3514.36.7.704

45. Spink KS, Longhurst K. Cognitive strategies and swimming performances: An exploratory study. Aust J Sci Med Sport. 1986;18(2):9–13.

46. Theodorakis Y. Effects of self-efficacy, satisfaction, and personal goals on swimming performance. Sport Psychol. 1995;9(3):245–53.

47. Thompson RW, Kaufman KA, De Petrillo LA, et al. One year follow-up of Mindful Sport Performance Enhancement (MSPE) with archers, golfers, and runners. J Clin Sport Psychol. 2011;5(2):99–116.

48. Wanlin CM, Hrycaiko DW, Martin GL, et al. The effects of a goal-setting package on the performance of speed skaters. J Appl Sport Psychol. 1997;9(2):212-28. doi:10.1080/10413209708406483

49. Weinberg R, Bruya L, Jackson A. Goal setting and competition: A reaction to Hall and Byrne. J Sport Exerc Psychol. 1990;12(1):92–7.

50. Weinberg RS, Gould D, Jackson A. Cognition and motor performance: Effect of psyching-up strategies on three motor tasks. Cogn Ther Res. 1980;4(2):239-45. doi:10.1007/BF01173655

51. Williams KD, Nida SA, Baca LD, et al. Social loafing and swimming: Effects of identifiability on individual and relay performance of intercollegiate swimmers. Basic Appl Soc Psych. 1989;10(1):73-81. doi:10.1207/s15324834basp1001_7

52. Wrisberg CA, Franks BD, Birdwell MW, et al. Physiological and psychological responses to exercise with an induced attentional focus. Percept Mot Skills. 1988;66(2):603-16. doi:10.2466/pms.1988.66.2.603
