# Supplementary material for: Psychological Determinants of Whole-Body Endurance Performance
Source: Sports Med. 2015 Mar 15;45(7):997–1015. doi: 10.1007/s40279-015-0319-6 (PMC4473096; doi:10.1007/s40279-015-0319-6)
Supplement: Supplementary file 3 — Supplementary material 3 (DOCX 78.7 kb) [file 40279_2015_319_MOESM3_ESM.docx]

Psychological Determinants of Whole-Body Endurance Performance

*Sports Medicine*

Alister McCormick (🖂), Carla Meijen, and Samuele Marcora

Endurance Research Group, University of Kent
E-mail: [am801@kent.ac.uk](mailto:am801@kent.ac.uk)

| **Electronic Supplementary Material Table S2**. Descriptive overview of practical intervention studies | | | | | | | |
| --- | --- | --- | --- | --- | --- | --- | --- |
| Intervention category | Study | Participant information | | Design overview | Exercise mode and performance variable | Intervention information | Effect on endurance performance |
| Association and dissociation | Morgan et al. [54] | 27 males serving in the army (age = 22.3 ± 0.4). | | Pretest-posttest design with a randomised control group (pre = 1  post = 1). | Walking  Exercise time in an incremental test. | Dissociative cognitive strategy (D). Ps were instructed to concentrate on and chase a spot in front of them and to repeat the word “down” with each leg movement. | Endurance time was greater in the D group (M = 21.5 min) than the control  (M = 14.5 min). More Ps improved in the D group  (*p* < .02). Δ = 1.06. |
|  | Okwumabua  et al. [47] | 31 university students from running fitness classes (f = 20,  m =11, M age = 21.4). | | Pretest-posttest design with two experimental groups and a control. Classes were randomly assigned as a group (pre = 1, post = 2). | Running  Performance time in a 1.5-mile run on a track. | Association (A) or dissociation (D).  A = monitoring bodily signals.  D = focusing on task-irrelevant objects and repeating a rhythmic phrase. Verbal instructions were given on a group basis. The first set of instructions lasted ten minutes, and they were paraphrased during two additional weekly sessions. | All groups improved  (*p* < .01) from the second to third performance (A = 9.5% / 1.32 min, D = 5.8%, placebo = 5.4%). A significant difference was not found between conditions. A Δ = 0.46. D Δ = 0.88. |
|  | Saintsing  et al. [56] | 50 university students (f = 19,  m = 31). | | Pretest-posttest design with three experimental groups and a control. Ps were matched before assignment (pre = 2, post = 2). | Running  Performance time in a 1.5-mile run on a track. | Association (A), dissociation (D), or psyching up (PU). A = task-specific thoughts including technique. D = task-irrelevant thoughts and repeating the word “down” with each stride. PU = self-chosen “firing up” method. Ps were given instructions as a group, and Ps had five minutes to mentally prepare before each run. A and D were practised during training runs. | The A group improved  (M = 58.3 s) to a greater extent (*p* < .05) than those in the D (M = 39.5 s), PU  (M = 37.9 s), or control groups (M = 26.8 s). |
|  |  |  | |  |  |  |  |
| **Table S2** continued | | | | | | | |
| Intervention category | Study | Participant information | | Design overview | Exercise mode and performance variable | Intervention information | Effect on endurance performance |
| Association and dissociation (continued) | Scott et  al. [58] | 9 rowers from a university rowing club (f = 5, m = 4, age = 20.2 ± 1.9). | | Single-subject, multiple-baseline design across participants. Ps were randomly assigned one of three interventions. Ps performed 10 trials. | Rowing  Distance rowed in 40 minutes on an ergometer. | Association or dissociation. Ps listened to an audio tape of a coxswain (association, A), watched a videotape of rowing races (dissociation, DV), or listened to pop music (dissociation, DM) during performance. | The A group demonstrated the greatest improvement in performance (M = 3.8% / 336 m). The DV (M = 1.3%) and DM (0.8%) groups also improved.  A Δ = 6.58, PND = 100%. DV Δ = 1.63, PND = 92%. DM Δ = 0.57, PND = 30%. |
|  | Weinberg et al. [57] Study 1 | 60 males from university conditioning classes. | | Between-subjects design. Ps were matched and assigned to one of three experimental conditions or a control. | Running  Distance ran in 30 minutes on a track. | Association (A), dissociation (D), or positive self-talk (S) during performance. A = monitoring bodily signals. D = pleasant, task-irrelevant thoughts. P = self-encouragement. Ps were given strategy instructions immediately before performing. | There was not a significant difference in the distance ran by Ps in the A, D, S, or control conditions. |
| Goal setting | Tenenbaum  et al. [59] | 28 female, secondary-school, cross-country runners (age = 14.6 ± 1.2). | | Pretest-posttest design with three experimental groups and no control. Ps were assigned by block randomisation (pre = 1, post = 4). | Running  Performance time in a  2.3 km run on a road course. | Assignment of an easy, challenging, or unrealistic combination of short-term and long-term goals (5%, 10%, or 15% improvement in four weeks with weekly targets). Goals were private and assigned verbally on an individual basis. | Each group’s best post-intervention performance was faster (*p* < .02) than baseline (M = 7.8%). Improvements did not significantly differ between groups. Combined ∆ (final performance) = 0.36. |
|  |  |  | |  |  |  |  |
| **Table S2** continued | | | | | | | |
| Intervention category | Study | Participant information | | Design overview | Exercise mode and performance variable | Intervention information | Effect on endurance performance |
| Goal setting (continued) | Theodorakis  et al. [51] | 40 university students (f = 23,  m = 17, age =  20.3 ± 2.1). | | Pretest-posttest design with a control group (pre = 1,  post = 1). | Cycling  Exercise time in an incremental test on an ergometer. | Goal setting and performance feedback. Ps set a specific goal (orally and in writing) for improved performance. Elapsed time was displayed during performance. | The goal setting group showed a greater increase  (*p* < .05) in endurance performance (M = 12.3% / 110.4 s) compared to the control (M = 1.9%).  Δ = 0.33. |
| Hypnosis | Jackson et al. [50] | 55 male university students (M age = 23.3). | | Pretest-posttest design. Ps were assigned to one of four experimental groups or a control (pre = 1, post = 1). | Running  Exercise time in an incremental test on a treadmill. | Post-hypnotic suggestion (PS). Ps were hypnotised and they then listened to a motivational passage. Interventions were delivered before performance by tape. | PS increased endurance time (*p* < .02) for high-susceptibility Ps (M = 15.9% / 57 s). The passage also increased endurance time in non-hypnotised Ps (M = 8.3%). These improvements did not significantly differ (*p* > .02).  High-susceptibility PS  Δ = 0.80. Low-susceptibility PS Δ = 0.13. |
|  | Lindsay et al. [44] | 3 nationally-ranked, competitive cyclists (f = 1, m = 2, age = 25.3 ± 5.9). | | Nonconcurrent single-subject, multiple-baseline design across participants. Ps raced 10 or 12 times. | Cycling  Points won in competitive road races. | Hypnosis was used to condition natural triggers experienced during races (e.g., feel of handlebars) to emotions associated with optimal performance. A four-stage intervention was delivered during one session. Ps practised daily using an audiotape of the session. | The number of points gained per race clearly increased for P1, sporadically increased for P2, and decreased for P3. ∆ = 1.85. PND = 52%. |
| **Table S2** continued | | | | | | | |
| Intervention category | Study | Participant information | Design overview | | Exercise mode and performance variable | Intervention information | Effect on endurance performance |
| Imagery | Burhans  et al. [60] | 65 university students (f = 29,  m = 36, age range = 17-22). | Pretest-posttest design with three experimental groups and a control. Ps were matched before random assignment (pre = 1, post = 2). | | Running  Performance time in a 1.5-mile run on a track. | Pre-performance imagery of perfect skill execution, successful performance outcomes, or both. Imagery was from an external perspective. Instructions were given on a group basis. Ps practised imagery for five to ten minutes before training runs and timed runs. | Experimental and control group performances were not significantly different  (*p* > .05) in the second of two posttests. |
|  | Post et  al. [55] | 4 competitive youth swimmers (f = 3,  m = 1, age =  15.5 ± 1.3). | Single-subject, multiple-baseline design across participants. Ps performed 12 trials. | | Swimming  Performance time in a 1,000-yard practice set. | Individualised imagery training and development of a personal imagery script. The intervention was delivered over nine sessions across three weeks. Ps were instructed to listen to the script three times a week, including once before timed performances. | The three Ps who adhered to the intervention's protocol demonstrated improved performance (M for these three Ps = 3.0% / 22.3 s).  Δ (all Ps) = 3.32.  PND (all Ps) = 75%. |
| Pre-performance statements | Donohue  et al. [33] | 6 female, collegiate, cross-country runners (age range =  18-21). | Within-subject design. A baseline performance preceded three counterbalanced experimental conditions. | | Running  Performance time in a  1 km outdoor run. | Instructional statements, motivational statements, or answering two questions (what were they thinking and feeling) during the warm-up. Ps selected the statements. Interventions were delivered by a research assistant. | Each intervention improved performance by 12 s to 19 s  (6-9%). Baseline and post-intervention performances did not significantly differ  (*p* > .05).  Instructional Δ = 2.11. Motivational Δ = 1.89. Questions Δ = 1.33. |
|  |  |  |  | |  |  |  |
| **Table S2** continued | | | | | | | |
| Intervention category | Study | Participant information | | Design overview | Exercise mode and performance variable | Intervention information | Effect on endurance performance |
| Pre-performance statements (continued*)* | Donohue  et al. [61] | 90 high-school distance runners  (f = 41, m = 49, age = 15.7 ± 1.1). | | Pretest-posttest design with two experimental groups and a control. Ps were matched before random assignment (pre = 1, post = 1). | Running  Performance time in a 1-mile run on a track. | Pre-performance motivational group intervention. Ps shouted a chosen motivational statement during group exercises. The intervention lasted 20 minutes. | Ps in the motivational intervention showed greater improvement (*p* < .001) in performance (M = 1.4% / 5 s) compared to those in yoga (M = 1 s) and control conditions (M = -1 s).  Δ = 0.10. |
|  | Miller and Donohue [46] | 90 high-school distance runners  (f = 45, m = 45, age = 16.2 ± 1.1). | | Pretest-posttest design with two experimental groups and a control. Ps were matched before random assignment (pre = 1, post = 1). | Running  Performance time in a  1.6 km run on a track. | Motivational and instructional statements delivered through headphones during the three minutes preceding performance. Ps selected the statements. | Motivational and instructional statements  (M = 2.3% / 8 s) and a self-selected song (M = 1.3%) improved endurance performance (*p* < .001).  Statements Δ = 0.09. |
|  | Weinberg  et al. [62] | 81 collegiate, cross-country runners  (f = 40, m = 41, age = 19.5 ± 1.3). | | Pretest-posttest design with six experimental groups and no control. Ps were assigned using matching procedures (pre = 1, post = 1). | Running  Performance time in a 1-mile run. | Motivational statements, instructional statements, or both (MI) were self-chosen or assigned. Ps listened to statements (read by the experimenter) on a CD for three minutes before performing. Ps believed that their coach chose the assigned statements. | Three groups improved their performance (*p* < .01). MI self-chosen statements led to the greatest improvement (M = 3.0% / 10 s). The type of statements or who assigned them did not consistently predict performance.  Δ range = 0.03 - 0.18. |
|  |  |  | |  |  |  |  |
| **Table S2** continued | | | | | | | |
| Intervention category | Study | Participant information | | Design overview | Exercise mode and performance variable | Intervention information | Effect on endurance performance |
| Psychological skills training (PST) package | Barwood  et al. [48] | 18 males (PST age = 23 ± 3, control age = 28 ± 5). | | Pretest-posttest design with a control group. Ps were matched before random assignment (pre = 2, post = 1). | Running  Distance ran during 90 minutes in 30°C heat in a climate chamber. | PST package to meet the demands of exercising in the heat. Four one-hour PST sessions were delivered in the four days preceding performance (goal setting, arousal regulation, mental imagery, and positive self-talk). | The PST group ran farther (M = 8% / 1.15 km) after receiving the intervention  (*p* < .05). The control group ran similar distances in each trial. ∆ = 0.54. |
|  | Patrick and Hrycaiko [39] | 3 triathletes of varying ability and 1 national-level runner (m = 4, age range = 25-37). | | Single-subject, multiple-baseline design across participants. Ps performed 11 trials. | Running  Performance time in a  1.6 km run on a track. | PST package delivered on an individual basis over three days (relaxation, imagery, self-talk, and goal setting). Skills were presented in a self-teaching workbook that contained reading and exercises. The first two sessions lasted 90 minutes, and a third session was dedicated to answering questions. | All Ps improved their performance following the intervention. ∆ = 4.22.  PND = 83%. |
|  | Sheard and  Golby [45] | 36 national-level swimmers (f = 23, m = 13, age = 13.9 ± 2.0, age range = 10-18). | | Pretest-posttest design without a control group. Ps’ best competitive performance times were obtained pre-, post-, and one-month post-intervention. | Swimming  Competition performance times for different strokes and distances. | PST program. Five weekly sessions were conducted on a one-to-one basis (goal setting, visualisation, relaxation, concentration, and thought stopping). Each session was personalised and lasted 45 minutes. | Performance time was faster (*p* < .05) in one out of five endurance events post-intervention. Performance times were faster (*p* < .05) in two endurance events one-month post-intervention.  Δ (post) = 0.03.  Δ (one-month post) = 0.28. |
|  |  |  | |  |  |  |  |
| **Table S2** continued | | | | | | | |
| Intervention category | Study | Participant information | | Design overview | Exercise mode and performance variable | Intervention information | Effect on endurance performance |
| PST package (continued) | Thelwell and Greenlees [40] | 5 male members of a gymnasium (age = 24.2 ± 4.6). | | Single-subject, multiple-baseline design across participants. Ps performed 10 trials. | Gymnasium triathlon  (2 km row,  5 km cycle, 3 km run)  Performance time. | PST package delivered on a one-to-one basis over four consecutive days (goal setting, relaxation, imagery, and self-talk). Each session lasted up to one hour and included education, workbook exercises, and homework. | All Ps improved their performance (M = 32.6 s) following the intervention.  ∆ = 2.80. PND = 81%. |
|  | Thelwell and Greenlees [41] | 4 male members of a gymnasium (age range = 19-21). | | Single-subject, multiple-baseline design across participants. Ps performed 10 trials. | Gymnasium triathlon  Performance time. | See Thelwell and Greenlees [40]. | All Ps improved their performance (M = 7.5% / 81 s) following the intervention. ∆ = 4.29.  PND = 90%. |
| Relaxation and biofeedback | Caird et  al. [63] | 7 sub-elite, competitive, long-distance runners. | | Within-subject design (pre = 2, post = 1). | Running  Peak velocity in a treadmill incremental test. | Biofeedback, progressive muscular relaxation, and centering to improve running economy. Ps attended training three times each week for six weeks (13-25 minutes per visit). Ps also practised lowering their heart rate each day using centering. | Biofeedback and relaxation improved running economy. Peak running velocity was unchanged. |
|  |  |  | |  |  |  |  |
| **Table S2** continued | | | | | | | |
| Intervention category | Study | Participant information | | Design overview | Exercise mode and performance variable | Intervention information | Effect on endurance performance |
| Self-talk | Barwood  et al. [53] | 14 recreationally-active males  (age = 19 ± 1). | | Pretest-posttest design with a control group. Ps were matched before assignment  (pre = 3, post = 1). | Cycling  Performance time in a  10 km time trial on an ergometer. | One-hour classroom session with a structured workbook. Ps identified their used negative self-talk statements and chose motivational statements to counter them with during each 2 km section. Ps rehearsed statements during the days and moments preceding each time trial. | Motivational self-talk improved time-trial performance (M = 3.75%,  *p* < .01). Neutral self-talk did not (M = -1.30%,  *p* = .312). ∆ = 0.39. |
|  | Blanchfield  et al. [49] | 24 recreationally-trained individuals  (f = 9, m = 15, age = 24.6 ± 7.5). | | Pretest-posttest design with a randomised control group (pre = 1,  post = 1). | Cycling  Time to exhaustion on an ergometer. | Two-stage self-talk intervention delivered over two weeks using a workbook. Stage 1 = introduction to self-talk and selection of four motivational self-talk statements.  Stage 2 = using self-talk during three or more exercise sessions. | Time to exhaustion increased (*p* ˂ .05) in the self-talk group (M = 17.9%  / 114 s) but not in the control (-2.5%). ∆ = 0.66. |
|  | Hamilton  et al. [64] | 9 university students (f = 3, m = 6, age = 20.9 ± 2.9). | | Single-subject, multiple-baseline design across participants. Ps were randomly assigned to one of three interventions. Ps performed 10 trials. | Cycling  Total work during 20 minutes of ergometer cycling. | Self-regulated positive (SP), assisted positive (AP), or assisted negative (AN) self-talk. In the SP condition, Ps were instructed in how to use positive statements during performance. In the assisted conditions, Ps were encouraged to use statements that were delivered by audiotape. | The AP group demonstrated the greatest performance improvement (M = 32.0%). The SP (M = 23.4%) and AN (11.0%) groups also increased total work.  AP Δ = 4.56. PND = 100%.  SP Δ = 2.35. PND = 100%.  AN Δ = 0.48. PND = 37%. |
| **Table S2** continued | | | | | | | |
| Self-talk (continued) | Rushall and Shewchuk [65] | 6 nationally-ranked swimmers (f = 4,  m = 2). | | Within-subject design. Experimental and control trials were included within each performance (e.g., alternation every 100 m during the 400 m swim). | Swimming  Training times for two 400 m swims and a set of eight 100 m repeats. | Positive thinking (PT, e.g., “I’m doing great”), mood words (MW, e.g., “blast”), or task-relevant thinking (TT, e.g., “elbows up”) during performance. Ps were given an instruction sheet with explanations and examples, and they practised during two training sessions. | For the 400 m effort swims and the eight 100 m repeats, times were faster in the PT (M = 1.4% and 2.1%), MW (M = 3.1% and 2.3%) and TT (M = 3.1% and 2.5%) conditions compared to the control (*p* < .05). |
| *f* number of female participants, *m* number of male participants, *M* mean, *P(s)* participant(s), *PND* mean percentage of non-overlapping data points, *post* number of post-intervention performances, *pre* number of pre-intervention performances, *±* mean ± standard deviation,  *Δ* effect size (Glass’s delta) | | | | | | | |

| **Electronic Supplementary Material Table S3**. Descriptive overview of additional psychological determinant studies | | | | | | |
| --- | --- | --- | --- | --- | --- | --- |
| Psychological determinant | Study | Participant information | Design overview | Exercise mode and performance variable | Experimental manipulation | Effect on endurance performance |
| Co-participation | Bath et  al. [73] | 11 club-level male athletes  (age = 33 ± 8). | Within-subject design. T2-T4 were randomised.  T1 = Self-paced  T2 = Running behind co-participant  T3 = Running ahead of co-participant  T4 = Running alongside  T5 = Self-paced | Running  Performance time in a  5 km time trial on a track. | A second runner who ran at a similar pace and maintained a set distance. Ps were not instructed to compete against the second runner. | Performance times were not significantly different between trials (*p* = .208). Mean Δ = - 0.07 |
|  | Williams et al. [74] | 15 competitive male cyclists (median  age = 34). | Within-subject design. T3-T4 were randomised and counterbalanced.  T1 = Familiarisation  T2 = One avatar (P progress).  T3 = Two avatars (P + competitor progress)  T4 = Feedback of distance covered | Cycling  Performance time in a 16.1 km time trial on an ergometer. | An avatar representing the previous performance of another cyclist of similar ability. Ps were not instructed to compete against the avatar. The competitor’s avatar was actually a representation of their best performance in T1 or T2. | Ps performed faster in the co-participation trial (27.8 ± 2.0 min) than T2 (28.7 ± 1.9 min, *p* = .001) and T4 (28.4 ± 2.3 min, *p* = .067).  Δ (versus T2) = 0.47.  Δ (versus T4) = 0.26. |
| Efficacy strength (ES) | Miller [82] | 84 regional- or national-level swimmers  (f = 42, m = 42, age = 14.4 ± 3.0, age range =  10-22). | Between-subjects design. Random assignment with matching. | Swimming  Performance time in a  200 m medley. | Assignment of a goal time slower (high ES) or faster (low ES) than their personal best. | Ps with high ES performed better (relative to their personal-best times) than Ps with low ES, independent of skill level  (*p* < .001). |
| **Table S3** continued | |  |  |  |  |  |
| Psychological determinant | Study | Participant information | Design overview | Exercise mode and performance variable | Experimental manipulation | Effect on endurance performance |
| Emotion suppression | Wagstaff [81] | 19 club-,  national-, or international-level swimmers, runners, or rowers (f = 9, m = 10, age = 21.1 ± 1.6). | Within-subject design. T2-T4 were randomised and counterbalanced.  T1 = Familiarisation  T2 = Control  T3 = Suppression  T4 = Nonsuppression | Cycling  Performance time in a  10 km time trial on an ergometer. | Pre-performance intervention. Ps were asked to conceal their emotions whilst watching a disgusting video. | Ps performed slower (*p* = .02) in the suppression condition (18.4 ± 1.1 min) than the nonsuppression (18.0 ± 1.2) and control conditions (17.8 ± 1.1 min).  Δ (versus nonsuppression) = 0.34.  Δ (versus control) = 0.56. |
| Experimenter characteristics | Bubb et al. [79] Study 1 | 80 college students (f = 40, m = 40). | Between-subjects design. | Running  Exercise time in an incremental test on a treadmill. | The experimenter was the same or opposite sex of the participant, and they were categorised as being the same or different race. | An interaction effect (*p* = .029) indicated that experimenter sex could influence endurance performance, depending on the sex and race of participants. |
|  | Bubb et al. [79] Study 2 | 20 adults (f = 10, m = 10). | Between-subjects design. | Running  Exercise time in an incremental test on a treadmill. | Participants were either friends with the researcher or they were unfamiliar with one another. | There was not a significant difference in performance between groups (*p* > .10). |
| Financial incentive | Hulleman et al. [71] | 7 male cyclists who competed at regional level (age = 32.1 ± 10.8). | Within-subject design (pre = 3, post = 1). | Cycling  Performance time in a  1.5 km time trial on an ergometer. | Monetary incentive ($100) to beat their best time by more than one second. | There was not a significant difference in performance time or total power output (*p* > .05) following the incentive (T2 time = 133.1 ± 2.1 s, T3 = 134.1 ± 3.4s, T4 = 133.6 ± 3.0 s). |
| **Table S3** continued | |  |  |  |  |  |
| Psychological determinant | Study | Participant information | Design overview | Exercise mode and performance variable | Experimental manipulation | Effect on endurance performance |
| Head-to-head competition | Corbett et  al. [67] | 14 male regular exercisers (age = 19 ± 1). | Within-subject design. T4 and T5 were counterbalanced.  T1-3 = Familiarisation  T4 = Exercising alone  T5 = Simulated head-to-head competition. | Cycling  Performance time in a  2 km time trial on an ergometer. | Competition against their best familiarisation trial, disguised as head-to-head competition against a competitor of similar ability. | Ps performed faster  (*p* = .021) in the competitive condition (T5 = 184.6 ± 6.2 s) than when they performed alone  (T4 = 188.3 ± 9.5 s). Δ = 0.39. |
|  | Higgs [66] | 20 female university students. | Pretest-posttest design without a control group (pre = 1, post = 1). | Running  Time to exhaustion on a treadmill. | Head-to-head competition against a “matched” competitor (the competitor had superior endurance). | Ps ran for longer (*p* < .01) in the competitive condition  (M = 299.4 s) than the self-motivated condition (M = 270.9 s), independent of their level of competitiveness. |
|  | Peveler and Green [68] | 8 recreational or trained male cyclists (age =  39 ± 7). | Within-subject design. T3 and T4 were counterbalanced.  T1 = Familiarisation  T2 = Improve ranked position  T3/T4 = Competition (chasing or leading). | Cycling  Performance time in a  20 km time trial on an ergometer. | Head-to-head competition against their closest competitor in a chasing or leading position. | Performance times were not significantly different (*p* > .05) when Ps were trying to improve their ranked position (33.8 ± 1.4 min), chasing (33.5 ± 1.3 min), and leading (33.8 ± 1.6 min).  Chasing (compared to T2)  Δ = 0.23. Leading Δ = 0.03. |
|  | Wilmore [31] | 22 male university students (age = 22.3 ± 3.54). | Within-subject design. T2 and T3 were counterbalanced.  T1 = Control  T2 = Competition  T3 = Control. | Cycling  Time to exhaustion on an ergometer. | Head-to-head competition against a matched participant, as well as their previous performance time. | Time to exhaustion and total work were greater (*p* < .05) in the competitive trial (T2 time = 457 ± 233 s) than when Ps performed alone (T3 = 379 ± 180 s). Δ = 0.41. |
| **Table S3** continued | | | | | | |
| Psychological determinant | Study | Participant information | Design overview | Exercise mode and performance variable | Experimental manipulation | Effect on endurance performance |
| Mental fatigue | MacMahon et al. [76] | 20 experienced runners (f = 2,  m = 18, age =  25.4 ± 3.2). | Within-subject design. Order of conditions was assigned randomly. | Running  Performance time in a  3 km time trial on an indoor track. | Pre-performance intervention. Demanding 90-minute cognitive task (replication of Marcora et al. [75]). | Completion times were slower  (*p* = .009) in the mental fatigue condition (12:11 ± 0:54) compared with the control (11:58 ± 0:48).  Δ = 0.27. |
|  | Marcora et  al. [75] | 16 adults involved in regular aerobic training (f = 6,  m = 10, age =  26 ± 3). | Randomised, controlled, crossover experimental design. | Cycling  Time to exhaustion on an ergometer. | Pre-performance intervention. Demanding 90-minute cognitive task that requires sustained attention, working memory, response inhibition, and error monitoring. | Time to exhaustion was lower  (*p* *=* .003) in the mental fatigue condition (640 ± 316 s) than the control condition (754 ± 339 s). Δ = 0.34. |
|  | Pageaux et  al. [77] | 12 physically-active adults  (f = 4, m = 8, age = 21 ± 1). | Randomised crossover experimental design with a familiarisation trial. | Running  Performance time in a  5 km time trial on a treadmill. | Pre-performance intervention. Demanding 30-minute cognitive task with or without response inhibition. | Mental exertion involving response inhibition impaired  (*p* = .008) time-trial performance (24.4 ± 4.9 min) compared to mental exertion without inhibition (23.1 ± 3.8 min). Δ = 0.34. |
| Priming | Blanchfield et al. [52] Experiment 1 | 13 recreationally-trained adults  (f = 6, m = 7, age range = 18-23). | Randomised and counterbalanced crossover experimental design. | Cycling  Time to exhaustion on an ergometer. | Subliminally-presented visual cues relating to affect (happy or sad faces). Cues were presented during performance. | Ps cycled for 12% longer (*p* = .04) when subliminally primed with happy faces than sad faces.  Cohen’s *d* = 0.26. |
| **Table S3** continued | | | | | | |
| Psychological determinant | Study | Participant information | Design overview | Exercise mode and performance variable | Experimental manipulation | Effect on endurance performance |
| Priming (continued) | Blanchfield et al. [52] Experiment 2 | One male regional-level endurance athlete (age = 22). | Single-subject blocked randomisation tests design (12 experimental trials). | Cycling  Time to exhaustion on an ergometer. | Subliminally-presented word cues relating to action (action, go, lively, energy) or inaction (stop, toil, sleep, tired). Cues were presented during performance. | Subliminal priming with action words increased (*p* = .04) time to exhaustion by 18.3% compared to inaction words. |
|  | Hodgins et  al. [78] | 41 collegiate rowers (f = 24,  m = 17). | Between-subjects design with random assignment. | Rowing  Performance time in a  2 km time trial on an ergometer. | To prime autonomy, control, or impersonal motivation orientations, Ps used five words to construct grammatically-correct sentences. | Autonomy-primed rowers performed faster than control-primed and impersonally-primed rowers (*p* = < .05). |
| Talking | Franks and Myers [80] Study 1 | 16 college students (f = 8,  m = 8). | Within-subject design. Order of conditions was assigned randomly. | Running  Exercise time in an incremental test on a treadmill. | Answering questions about physical activity habits during performance. | There was not a significant difference (*p* > .10) in time to exhaustion between the talking (10.9 ± 3.1 min) and control  (11.2 ± 3.1 min) conditions.  Δ = -0.10. |
| **Table S3** continued | | | | | | |
| Psychological determinant | Study | Participant information | Design overview | Exercise mode and performance variable | Experimental manipulation | Effect on endurance performance |
| Verbal encouragement | Chitwood et al. [70] | 26 university students (f = 13,  m = 13, Type A age = 21.7 ± 0.5, Type B age =  21.7 ± 0.4). | Within-subject design. Order of conditions was assigned randomly. | Running  Exercise time in an incremental test on a treadmill. | Verbal encouragement during performance. | Type Bs ran for longer (*p* < .05) in the encouragement condition  (12.5 ± 5.0 min) than the control condition (10.8 ± 3.8 min).  Δ = 0.45. Performance times for Type As were similar in the encouragement (12.7 ± 4.2 min) and control (12.6 ± 3.7 min) conditions (*p* > .05). Δ = 0.03. |
|  |  |  |  |  |  |  |
|  | Moffatt et  al. [69] | 14 inter-collegiate cross-country runners and 14 non-athletes  (f = 8, m = 20, athlete age = 21.8 ± 1.4, non-athletes =  23.2 ± 4.7). | Within-subject design. Order of conditions was assigned randomly. | Running  Exercise time in an incremental test on a treadmill. | Verbal encouragement during performance. | Competitive runners ran for longer  (*p* < .05) in the encouragement condition (19.7 ± 1.2 min) than the control condition (16.7 ± 1.1 min).  Δ = 2.73. Non-athletes also ran for longer (*p* < .05) in the encouragement condition  (14.3 ± 0.9 min) than the control (12.4 ± 1.2 min). Δ = 1.58. |
|  | Viru et  al. [72] | 14 male  university-level endurance athletes (age range =  19-23). | Within-subject design. Order of conditions was assigned randomly. | Running  Exercise time in an incremental test on a treadmill. | Monetary incentives to better their own time and the times of others, as well as strong encouragement. | Ps ran for longer (*p* < .05) in the competitive condition  (1,222 ± 100 s) than the control condition (1,173 ± 121 s).  Δ = 0.40. |
| *f* number of female participants, *m* number of male participants, *M* mean, *P(s)* participant(s), *post* number of post-intervention performances, *pre* number of pre-intervention performances, *T* trial, *Type A / B* Type A / B personality participants, *±* mean ± standard deviation, *Δ* effect size (Glass’s delta) | | | | | | |
