# Supplementary material for: Psychological Determinants of Whole-Body Endurance Performance
Source: Sports Med. 2015 Mar 15;45(7):997–1015. doi: 10.1007/s40279-015-0319-6 (PMC4473096; doi:10.1007/s40279-015-0319-6)
Supplement: Supplementary file 4 — Supplementary material 4 (DOCX 16.6 kb) [file 40279_2015_319_MOESM4_ESM.docx]

Psychological Determinants of Whole-Body Endurance Performance

*Sports Medicine*

Alister McCormick (🖂), Carla Meijen, and Samuele Marcora

Endurance Research Group, University of Kent
E-mail: [am801@kent.ac.uk](mailto:am801@kent.ac.uk)

Electronic Supplementary Material Appendix S1. Keywords included in database searches.

Keywords Included in Database Searches

**Words meaning “endurance” and direct measures of endurance performance (*n* = 33)**

Endurance OR ultra-endurance OR “exercise tolerance” OR “exercise resilience” OR “work capacit*” OR “time-to-exhaustion” OR “time to exhaustion” OR “time to fatigue” OR “time-to-fatigue” OR “time trial*” OR “time-trial*” OR “performance time*” OR “performance distance*” OR “total power” OR “power output” OR “peak power” OR “maximum power” OR “maximal power” OR “exercise performance” OR "exercise time" OR "exercising time" OR “treadmill time” OR “distance ran” OR “distance cycled” OR “distance swam” OR “distance rowed” OR "volitional exhaustion" OR "voluntary exhaustion" OR “physical performance” OR “peak velocit*” OR “peak treadmill velocit*” OR “peak speed” OR “peak treadmill speed”

**Physiological dependent variables measured during endurance performance (*n* = 16)**

Economy OR efficiency OR “VO2” OR “V O2” OR “VO 2” OR "VO2max” OR "[Vdot]O2" OR "[Vdot] O2" OR “VO2peak” OR “maximal aerobic” OR “maximum aerobic” OR “maximal oxygen” OR “maximum oxygen” OR “peak oxygen” OR pacing OR pace

**Running keywords (*n* = 21)**

Run OR runner OR runners OR running OR cross-country OR “cross country” OR “fitness test” OR “beep test” OR “beep-test” OR “bleep test” OR “bleep-test” OR “pacer test” OR “pacer-test” OR “Leger test” OR “Leger-test” OR “shuttle run” OR “shuttle-run” OR marathon OR marathoner OR ultra-marathon OR ultra-marathoner

**Cycling keywords (*n* = 8)**

Cycle OR cyclist OR cyclists OR cycling OR bicycle OR ergometer OR bike OR biking

**Swimming keywords (*n* = 4)**

Swim OR swimmer OR swimmers OR swimming

**Rowing keywords (*n* = 4)**

Row OR rower OR rowers OR rowing

**Skiing keywords (*n* = 4)**

Ski OR skiing OR skier OR skiers

**Canoeing keywords (*n* = 4)**

Canoe OR canoer OR canoers OR canoeing

**Kayaking keywords (*n* = 4)**

Kayak OR kayaker OR kayakers OR kayaking

**Multi-leg sport keywords (*n* = 10)**

Triathlon OR triathlete OR triathletes OR biathlon OR biathlete OR biathletes OR duathlon OR duathlete OR duathletes OR Ironman

**Speedskating keywords (*n* = 8)**

Speedskate OR speedskater OR speedskaters OR speedskating OR “speed skate” OR “speed skater” OR “speed skaters” OR “speed skating”

**Race walking keywords (*n* = 12)**

Walk OR racewalk OR "race-walk" OR walking OR racewalking OR "race-walking" OR walker OR racewalker OR "race-walker" OR walkers OR racewalkers OR "race-walkers"

**Keywords relating to psychological states or manipulations (*n* = 78)**

psychologic* OR mental* OR cognitive* OR psychobiological* OR psychosocial* OR psychophysiolog* OR MST OR PST OR “performance enhancement” OR “performance-enhancement” OR strateg* OR technique* OR effective* OR “self-talk” OR “self talk” OR imagery OR visualisation OR visualization OR PETTLEP OR relaxation OR goal* OR “approach-goal*” OR "avoidance-goal*” OR “achievement-goal*” OR state OR optimism OR efficacy OR “self-efficacy” OR confidence OR self-confidence OR self-doubt OR doubt* OR motivation OR “self-determination” OR reward OR incentive* OR reinforcement OR encouragement OR compet* OR coaction OR “social facilitation” OR attention* OR focus OR concentration OR fear OR anxiety OR nervous* OR nerves OR arousal OR stress OR distress OR pressure OR mood OR affect OR emotion OR coping OR hypnosis OR hypnotic OR “post-hypnotic” OR “post hypnotic” OR RPE OR effort OR exertion OR fatigue OR belief OR “self-belief” OR feeling* OR association OR dissociation OR associative OR dissociative OR mindful* OR acceptance OR meditation OR meditating OR biofeedback OR bio-feedback OR talking
